# Physics‐Based Inverse Modeling of Battery Degradation with Bayesian Methods

**DOI:** 10.1002/cssc.202402336

**Published:** 2025-07-09

**Authors:** Micha C. J. Philipp, Yannick Kuhn, Arnulf Latz, Birger Horstmann

**Affiliations:** ^1^ Institute for Engineering Thermodynamics German Aerospace Center (DLR) Wilhelm‐Runge‐Straße 10 89081 Ulm Germany; ^2^ Theory of Electrochemical Materials Helmholtz Institute Ulm (HIU) Helmholtzstraße 11 89081 Ulm Germany; ^3^ Faculty of Natural Sciences Ulm University Albert‐Einstein‐Allee 11 89081 Ulm Germany

**Keywords:** Bayesian methods, inverse modeling, lithium‐ion batteries, machine learning, model selection, parameterizations, uncertainty quantifications

## Abstract

To further improve lithium‐ion batteries, a profound understanding of complex battery processes is crucial. Physical models offer understanding but are difficult to validate and parameterize. Therefore, automated machine‐learning methods are necessary to evaluate models with experimental data. Bayesian methods, e.g., Expectation Propagation + Bayesian Optimization for Likelihood‐Free Inference (EP‐BOLFI), stand out as they capture uncertainties in models and data while granting meaningful parameterization. An important topic is prolonging battery lifetime, which is limited by degradation, such as the solid‐electrolyte interphase (SEI) growth. As a case study, EP‐BOLFI is applied to parametrize SEI growth models with synthetic and real degradation data. EP‐BOLFI allows for incorporating human expertise in the form of suitable feature selection, which improves the parametrization. It is shown that even under impeded conditions, correct parameterization is achieved with reasonable uncertainty quantification, needing less computational effort than standard Markov Chain Monte Carlo methods. Additionally, the physically reliable summary statistics show if parameters are strongly correlated and not unambiguously identifiable. Further, Bayesian Alternately Subsampled Quadrature (BASQ) is investigated, which calculates model probabilities, to confirm electron diffusion as the best theoretical model to describe SEI growth during battery storage.

## Graphical abstract

Active parameterization of physics‐based models is essential to drive battery science. However, due to the model's complexity, parameterization is an ill‐posed problem with uncertainties in models and data. In this article, Bayesian methods are applied to physical degradation models to show sample‐efficient parameterization with uncertainty quantification. Physical conclusions, parameter dependencies, and model selection are derived using Bayesian statistics.

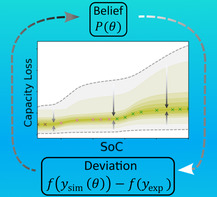

## 1 Introduction

Lithium‐ion batteries (LiBs) are of central importance for the transition to renewable energy sources, especially for the electrification of the transport sector. To meet the high requirements of the transport sector, such as high energy density and long service life, an in‐depth physical understanding of the complex battery processes is essential. The coupling of numerous complicated physicochemical effects makes gaining insights into the behavior of batteries challenging for the scientific community. However, the increasing capabilities of machine‐learning (ML) algorithms point to a possible way to tackle this problem.

ML references a broad class of numerical or statistical algorithms for automatized data analysis, pattern recognition, classification, and regression. With increased computational power, ML techniques have become more popular and researched. In battery research, there are many ML applications,^[^ ^1^ ^]^ e.g., early prediction of battery lifetime,^[^ ^2^ ^]^ material performance,^[^ ^3^ ^]^ battery state estimation,^[^ ^4^, ^5^ ^]^ increase in simulation speed,^[^ ^6^ ^]^ and parameterization of physics‐informed models.^[^ ^7^, ^8^, ^9^, ^10^, ^11^ ^]^ To make physical conclusions, analyzing available physical models and comparing them with experimental data utilizing ML methods is inevitable. Since this process is always accompanied by a lack of information, e.g., model uncertainty or measurement inaccuracies, a consistent uncertainty quantification (UQ) of the obtained results becomes increasingly important. The most natural incorporation of uncertainty is achieved by Bayesian algorithms, favoring them over other ML algorithms for these purposes. The capability of Bayes’ theorem, updating your prior knowledge with more available evidence, and the results in multivariate probability distributions make Bayesian algorithms a unique option in parameterization and inverse modeling. However, these algorithms usually need many simulated samples to obtain qualitatively good results,^[^ ^12^, ^13^, ^14^ ^]^ impeding the computationally heavy physical models. In this work, we show that using an improved Bayesian algorithm, Expectation Propagation + Bayesian Optimization for Likelihood‐Free Inference (EP‐BOLFI),^[^ ^15^ ^]^ enables a good parameterization for physical models with UQ, needing orders of magnitude less samples in contrast to common Markov Chain Monte Carlo (MCMC) methods. Further, the Bayesian Alternately Subsampled Quadrature (BASQ) model selection algorithm,^[^ ^16^ ^]^ based on Bayesian principles, is applied to identify the prevailing mechanism from a specific selection. As a case study for these Bayesian algorithms, we investigate modeling solid‐electrolyte interphase (SEI) growth, which limits the LiB lifetime.

Several degradation mechanisms^[^ ^17^, ^18^ ^]^ influence the lifetime of a LiB, e.g., the ongoing parasitic side reactions forming the SEI.^[^ ^19^ ^]^ The SEI is crucial for the functionality of the lithium‐ion battery by preventing direct contact between the electrode and the electrolyte.^[^ ^20^ ^]^ However, it does not ideally prevent side reactions as desired. The ongoing SEI growth consumes lithium ions and leads to capacity and performance losses.^[^ ^21^ ^]^ Despite long‐lasting research efforts, the fundamental physical processes involved in the ongoing SEI growth are not fully understood.^[^ ^22^, ^23^, ^24^ ^]^ Several mechanisms proposed in the literature aim to explain the continued SEI formation.^[^ ^25^, ^26^, ^27^ ^]^ One can classify these mechanisms roughly into two categories: the first predicts that the prevailing mechanism is solvent diffusion through the SEI, and the second category postulates the SEI to be blocking for solvent molecules but considers ongoing electron transport through the SEI by various effects. These mechanisms can describe the capacity fade due to ongoing SEI formation but have subtle differences in the dependence on the operating conditions. These differences allow the disentangling of SEI growth mechanisms with ML approaches.

**Figure 1:**
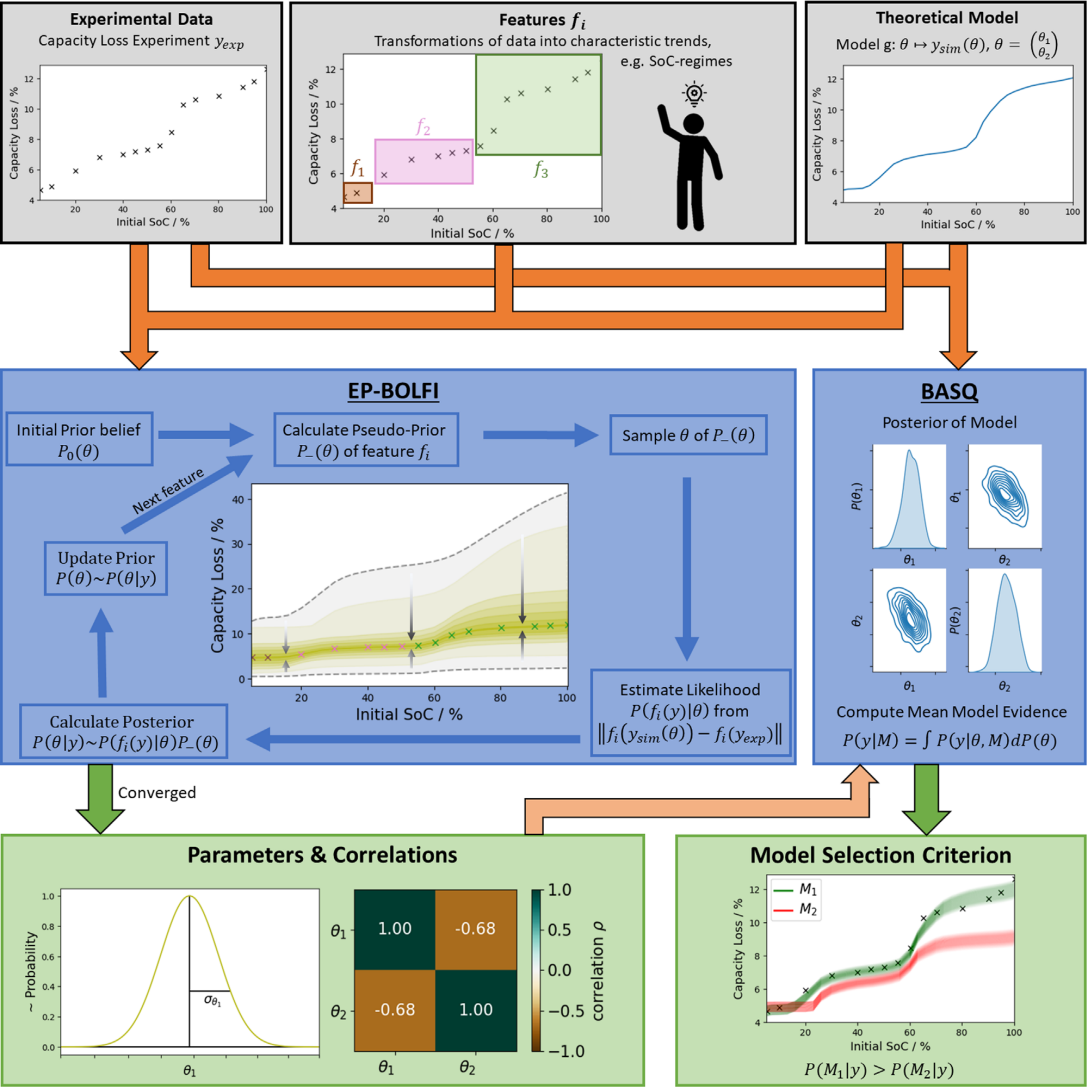
Simplified visualization of the workflow presented in this work applied to the case study of modeling the continuous growth of the SEI. The upper row shows the primary input for the algorithms, i.e., the experimental data $y_{\text{exp}}\;$ (see Section 3.3) and the model (e.g., a particular SEI growth mechanism, see Section 3.2). Human‐chosen features are input to EP‐BOLFI to improve its performance. The orange arrows indicate the input flows. The middle row shows the two Bayesian algorithms used in this work. EP‐BOLFI performs a multidimensional probabilistic fit iteratively (see Section 2.1). BASQ (see Section 2.2) determines the model posterior distribution and computes the mean model evidence. In the bottom row, the outputs are shown. From EP‐BOLFI, the optimal parameter values and corresponding (co‐)variances or correlations are obtained. To improve BASQ's performance, a preconditioned parameterization received by EP‐BOLFI is helpful as additional input, as indicated by the faint orange arrow. Applying BASQ for different models yields a model selection criterion, as the “better” model obtains a higher mean model evidence.

This work presents a workflow for analyzing physically derived models with advanced Bayesian methods. We apply these methods to the example of battery aging due to continuous SEI formation. We present the workflow in **Figure** **1**. The Bayesian algorithms (see Section 2) take the experimental data and physics‐based models (see Section 3) as input and yield a parameterization with UQ, parameter correlations, and a model selection criterion as results (see Section 4). We conclude in Section 5.

## 2 Bayesian Methods

In Bayesian statistics, the term probability is referred to certainty. In contrast to the usual frequentist approach, data points are considered fixed, and the parameters are uncertain. Instead of finding the perfect parameter value, the aim is to estimate the posterior certainty distribution of a desired parameter set *θ*, considering the prior knowledge and the data *y*. Bayes’ theorem states that given prior knowledge or assumption *P(θ)* can be updated with further knowledge or likelihood P(y|θ) , to obtain a posterior distribution P(θ|y)  by

(1) $$P(\theta |y)=\frac{P(y|\theta )P(\theta )}{P(y)}$$

where *P(y)* is the evidence of the data. In the following subsections, we introduce the EP‐BOLFI and BASQ algorithms, which use Bayes’ theorem and the Bayesian concept of probability.

### 2.1 Bayesian Optimization

The algorithm used in this work for Bayesian Optimization is called EP‐BOLFI, developed by Kuhn et al.^[^ ^15^ ^]^ This algorithm is utilized for inverse modeling and combines two algorithms: EP and BOLFI. This combination aims to maintain the advantages of Bayesian inference (BOLFI) while achieving a substantial reduction in needed simulation samples (by EP) compared to standard approaches.^[^ ^15^ ^]^

EP^[^ ^28^ ^]^ (see left blue box in Figure 1) introduces the approach of splitting the data into several features *f*_*i*_, and then propagating the gained information through every feature *f*_*j≠i*_, substantially reducing the needed simulation samples. The choice of features is highly flexible and can be adapted to different problems. A trivial example is segmenting the data into different coherent parts and considering the data points in one single part as one feature. However, experts can also incorporate physical knowledge about the system by choosing certain data transformations as features, e.g., physically motivated fit functions. The feature then consists of the parameters describing the transformation. A feature can be thought of as a point in a higher‐dimensional space. The resulting distance between this point for the simulated data and the point from the experimental data will be used for optimization. Therefore, it is essential to note that the selected transformations form the underlying landscape of a loss function in parameter space. Suitable choices can improve the results regarding noise stability, convergence speed, resulting uncertainty, and correct parameter identifiability.

BOLFI^[^ ^29^ ^]^ (see left blue box in Figure 1) is responsible for fitting the selected features one by one to the experimental data. Firstly, different parameter configurations θ  are drawn from the prior belief P(θ)  and simulated. In the second step, the distance between the selected feature for the simulated data $f_{i}(y_{\text{sim}}(\theta ))\;$ and the experimental data *f*_*i*_(*y*_exp_) is calculated. In the third step, a Gaussian process is trained on the parameter‐distance pairs ($\theta ,\text{log}\left(\left\|f_{i}\left(y_{\text{sim}}\left(\theta \right)\right)-f_{i}\left(y_{\text{exp}}\right)\right\|\right)\;$). Fourthly, transforming this Gaussian Process with

(2) $$P(y|\theta )\approx P(\text{log}(\|f_{i}(y_{\text{sim}}(\theta ))-f_{i}(y_{\text{exp}})\|)\leq \varepsilon )$$

whereby ε  is a certain threshold, yields the likelihood of the specific feature $f_{i}\;$. Fifthly, the product of the likelihood and the prior (see Equation 1) becomes evaluated by MCMC sampling. The result is the posterior distribution P(θ|y) , without the need to explicitly determine the evidence P(y) .

Often, the likelihood is assumed to follow a particular distribution, e.g., a normal distribution. However, this may differ for coupled battery mechanisms, which can generate a more complex manifold in the parameter space. Therefore, the Gaussian process is used as a flexible surrogate for the likelihood.

The posterior is reduced to a multivariate normal distribution for better interpretability and ease in the following use. Barthelmé et al.^[^ ^30^ ^]^ proved that in the case of a normal posterior, the EP procedure will converge to this exact posterior, and the reduction will not distort the result. A normal posterior is a good approximation, as identifiable problems tend to have a parabola‐shaped optimum. In the sense of a traceback, the likelihood of the specific feature can then be calculated backwards from Equation 1, also as a normal distribution. The subsequent EP step realizes the propagation of the gained information. The obtained parameters’ posterior distribution of feature $f_{i}\;$ enters as the prior belief for the next feature $f_{i+1}\;$. Besides, previously gathered information for this next feature ($f_{i+1}\;$) is removed from the prior. In this way, the prior belief contains only the information from the other already simulated features and/or the initial prior.

By iterating through the selected features (multiple times if needed), this procedure finally provides the overall posterior and feature‐specific likelihoods as multivariate ellipsoids in hyperspace, with the most likely parameterization as the means and the corresponding uncertainty through the covariance matrices. Since the feature‐specific likelihoods become updated during each iteration, we refer to the final likelihoods of the individual features as the feature‐specific posteriors.

### 2.2 Bayesian Quadrature

Bayesian Quadrature (BQ) is a method to approximate intractable integrals. In the algorithm BASQ, see right blue box in Figure 1) developed by Adachi et al.^[^ ^16^ ^]^ BQ is used to integrate the probability distribution P(y|θ,M)  of the data *y* given the parameters *θ* and model M over the parameter space of this model

(3) P(y|M)=∫P(y|θ,M)dP(θ)

With Bayes’ theorem, one can relate this to a model probability

(4) $$P(M|y)=\frac{P(y|M)P(M)}{P(y)}$$

Without further assumptions or information, all models enter with an equal prior probability P(M)  and the same normalizing data evidence P(y) , leaving P(y|M)  to be computed. Comparing the results for different models yields the Bayes factor

(5) $$K=\frac{P(M_{1}|y)}{P(M_{2}|y)}=\frac{P(y|M_{1})P(M_{1})P(y)}{P(y|M_{2})P(M_{2})P(y)}=\frac{P(y|M_{1})}{P(y|M_{2})}$$

Therefore, the model that achieves the highest value for the mean model evidence P(y|M)  is considered the “best” model to describe the data. Note that this quantity itself is a non‐normalized probability, only the comparison to a second model gives a significant meaning.

Considering the likelihood P(y|θ,M)  assigns a probability to a certain combination of model and parameter by weighting the distance to the data, this tends to prefer models with more available parameters to achieve the most accurate results. To avoid overfitting, we also investigate the effect of adding a penalty term to the likelihood, penalizing the number of parameters and their values (see S1, Supporting Information).

## 3 Theoretical and Experimental Section

### 3.1 Battery Cell Model

Physics‐informed battery models describe multiple coupled physicochemical effects as partial differential equations (PDE). Even though resolving the physical effects in a battery most accurately requires a microstructure‐resolved 3D description,^[^ ^31^ ^]^ numerically solving the system of PDEs in 3D is computationally heavy. Therefore, a trade‐off between physical accuracy and computation time has to be made. The common reduction in complexity is reducing the system's dimensionality by volume‐averaging. Thus, the Doyle–Fuller–Newman (DFN)^[^ ^32^ ^]^ p2D model has been developed, which captures accurately most battery effects. However, it is still computationally challenging, especially for ML applications requiring thousands of simulations. Further reduction in complexity by asymptotic analysis of the DFN yields the Single‐Particle Model (SPM) and SPM considering electrolyte effects (SPMe).^[^ ^33^ ^]^ In this work, we will use the SPM/SPMe description of a battery.

In the simple SPM picture, all particles in each electrode act equally, so we only resolve one single particle. At the surface of this particle the Li ions intercalate or deintercalate and radially diffuse inside due to concentration gradients. This description is reasonable in the case of vanishing currents and, therefore, is used in this work to simulate battery storage. The SPMe model considers additional effects in the electrolyte, which is computationally slightly more expensive but more accurate for small applied currents. In this work, we used the SPMe to simulate the cycling behavior of the battery cell. The nondimensional version of the governing equations of the SPMe, the model parameters, and the complementing initial and boundary conditions are summarized in S2, Supporting Information. The battery simulations were performed with PyBaMM.^[^ ^34^ ^]^

Commonly, the system is solved in time by initializing a certain battery state and giving the battery's current as input. The output contains all of the resolved battery states at different positions in space and time, in this work referred to as the simulated data $y_{\text{sim}}\;$. In this picture, we can describe the battery model as a function *g*, which takes the applied current profile I  and the battery parameters *θ* as input so that

(6) $$y_{\text{sim}}(t)=g(t,I,\theta )$$

For the case study pursued in this work (degradation by SEI formation), the most important simulated output is the battery's capacity loss (CL), which occurs due to SEI growth during battery operation/simulation and is described by the degradation models. For the parameterization, we assumed to have an already correct parameterized underlying battery cell and focused on the parameterization of the degradation models only.

### 3.2 Degradation Models

In a battery, many degradation effects take place simultaneously. One of the least understood is the formation of the SEI, which is considered the dominant degradation process during battery storage but also contributes to degradation during battery operation. The circumstances in which multiple theoretical SEI models exist depict this as an ideal case study to use ML methods to identify which mechanisms take place and contribute up to which degree. Therefore, this is the focus of this work.

The SEI is a passivating layer at the negative electrode of a battery formed by the reduction reactions of electrolyte molecules. This reaction irreversibly consumes Li ions, reducing the overall capacity and increasing the battery's internal resistance over time. To explain the observed growth of the SEI^[^ ^35^ ^]^ by ongoing reduction, a transport process of the reactants (electrons and solvent) to the reaction location has to take place. From a multiscale perspective, the literature^[^ ^25^, ^26^, ^27^ ^]^ proposes the following transport mechanisms for long‐term SEI growth: electron diffusion, electron conduction or migration, and solvent diffusion. In the following, we introduce these mechanisms briefly.

Electron diffusion (ED)^[^ ^25^, ^26^, ^36^, ^37^, ^38^, ^39^ ^]^ describes a diffusive transport of the electrons from the electrode through the SEI by hopping between localized states, e.g., lithium interstitials. At the interface between SEI and electrolyte these electrons are consumed in SEI formation reactions. How fast this reaction takes place depends on local quantities like the onset potential of the SEI formation reaction $\Phi_{0}\;$ and the electrical potential. Kolzenberg et al.^[^ ^26^ ^]^ investigated a reaction limitation in more detail and showed that this is important only for the early stages of SEI formation. Hence, we neglected the influence of the onset potential here and assumed instantaneous reactions once all reactants arrive at the reaction site, i.e., SEI formation reactions instantly consume all electrons. This leads to a vanishing concentration of electrons at the interface between SEI and electrolyte and a specific reference concentration at the electrode, which depends on the state of charge (SoC). Then, the diffusive transport of electrons that contribute to the SEI reaction is given by the following electron current density

(7) $$j_{\text{ED}}=\frac{c_{e^{-}}D_{e^{-}}F}{L_{\text{SEI}}}\text{exp}(-{\tilde{\eta }}_{\text{SEI}})$$

where $c_{e^{-}}\;$ is the reference concentration, $D_{e^{-}}\;$ is the diffusion constant of electrons through such localized states, *F* is the Faraday constant, and $L_{\text{SEI}}\;$ is the thickness of the SEI. ${\tilde{\eta }}_{\text{SEI}}\;$ is the SEI overpotential, which describes the amount of electrons available in the SEI at the electrode–SEI interface. This is given as

(8) $${\tilde{\eta }}_{\text{SEI}}=\frac{F}{RT}\left(\eta_{\text{int}}+U(\text{SoC})+\frac{\mu_{\text{Li,0}}}{F}\right)$$

where *R* is the universal gas constant, *T* is the temperature, $\mu_{\text{Li,0}}\;$ is the standard chemical potential of neutral lithium to lithium metal, U(SoC) is the open‐circuit voltage of the anode, and $\eta_{\text{int}}\;$ is the intercalation overpotential. For standard Butler–Volmer kinetics, this intercalation overpotential is defined by the following relation to the intercalation current density

(9) $$j_{\text{int}}=2j_{0}\text{sinh}\left(\frac{F}{2RT}\eta_{\text{int}}\right)$$

with the exchange current density $j_{0}$.

Another possible transport mechanism of electrons can occur due to a gradient in the electrical potential through the SEI. Combined with Ohms's law, this leads to a net current of electrons from the anode to the SEI–electrolyte surface. Depending on the origin of the gradient in the electrical potential, this can yield different current densities. One possibility, referred to as electron conduction (EC),^[^ ^22^, ^25^ ^]^ assumes that the step in the electrical potential between electrode and electrolyte follows a constant gradient over the thickness of the SEI during storage. Its current density is given by

(10) $$j_{\text{EC}}=\frac{\kappa (\Phi_{0}-U(\text{SoC}))}{L_{\text{SEI}}}$$

where κ  is the electron conductivity of the SEI, and $\Phi_{0}\;$ is the onset potential of the SEI formation reaction. The solvent becomes unstable at this potential and becomes reduced to form SEI compounds. Note that this only provides a current for SEI formation if $U(\text{SoC})<\Phi_{0}\;$. The leading critics of this mechanism address the conductivity of the SEI, which is considered to be an insulator in the relevant voltage regime,^[^ ^40^, ^41^ ^]^ and the assumption of a constant gradient in the electrical potential from the anode to the SEI–electrolyte interface. The electrical potential drops at the interfaces between electrode and SEI and between SEI and electrolyte, putting the assumption of a simple constant gradient over the whole thickness of the SEI into question.^[^ ^42^ ^]^

During battery operation, especially charging, the intercalation current of positively charged Li ions causes a change in the electrical potential at the electrode–SEI interface.^[^ ^26^ ^]^ The assumption that this electrical potential drops linearly toward the SEI–electrolyte interface, referred to as electron migration (EM), causes the following current density

(11) $$j_{\text{EM}}=\frac{c_{e}-D_{e}-F^{2}j_{\text{int}}}{2RT\kappa_{{\text{Li}}^{+}\text{,SEI}}}\text{exp}(-{\tilde{\eta }}_{\text{SEI}})$$

where $\kappa_{{\text{Li}}^{+}\text{,SEI}}\;$ is the lithium‐ion conductivity of the SEI.

Solvent diffusion (SD)^[^ ^43^, ^44^, ^45^, ^46^, ^47^ ^]^ assumes that the continuous transport of solvent limits the reduction reactions. One assumes a vanishing concentration of the solvent at the reaction site, again due to instantaneous reactions, and a constant solvent concentration in the bulk electrolyte. Then, the transport of the solvent is realized by diffusion, due to concentration gradients, through the SEI to the electrode reaction surface. Deriving a current density, in terms of electrons lost to SEI formation, this mechanism yields

(12) $$j_{\text{SD}}=\frac{c_{S}D_{S}F}{L_{\text{SEI}}}$$

where $c_{S}\;$ is the concentration of solvent molecules in bulk, and $D_{S}\;$ is the diffusion constant of the solvent molecules through the SEI. The physical credibility behind the process is also highly discussed, e.g., the solvent molecules have to be able to diffuse through the SEI pores, even though their reaction should close those.^[^ ^48^ ^]^

Despite decades‐long research efforts, the scientific community cannot fully explain the SEI growth, suggesting that the SEI is complex and possibly based on multiple coupled mechanisms. Single et al.^[^ ^25^ ^]^ and Köbbing et al.^[^ ^27^ ^]^ investigated SEI growth under storage conditions and found that electron diffusion best describes the SoC‐dependent trend in the experimental data. However, both works need an additional SoC‐independent capacity loss, whose origin is unclear. The SOC‐independent capacity loss is possibly due to an additional SEI growth mechanism, where only solvent diffusion matches this functionality. Therefore, we modeled SEI growth by combining SoC‐dependent mechanisms (electron diffusion or electron conduction) with solvent diffusion. With this knowledge, we investigated the following combinations as SEI models for battery storage. First, we labeled the combination of electron diffusion and solvent diffusion as the “Best Model” since previous work verified that it matches the experimental data. Second, we labeled the combination of electron conduction (with $\Phi_{0}=0.145\;$V) and solvent diffusion as the “Wrong Model” because the parameter value needed for $\Phi_{0}\;$ to capture the trends in the experimental data is unreasonable. Third, we considered the combination of electron diffusion, electron conduction, and solvent diffusion, which we labeled the “Overfitted Model” because it has more fit parameters than needed. For battery cycling, we additionally have to include electron migration. We labeled the combination of electron diffusion, solvent diffusion, and electron migration as the “Cycling Model”.

To model such combinations, we combined the derived current densities $j_{i}\;$ with i∈{ED,EC,EM,SD}  linearly to a total SEI current density $j_{\text{SEI}}=\sum j_{i}\;$, in case multiple transport mechanisms occur at a time. This current density was then incorporated into the battery model by assuming that the overall battery cell current density *j* consists of the intercalation current density $j_{\text{int}}\;$ and the SEI current density $j_{\text{SEI}}\;$ so that $j=j_{\text{int}}+j_{\text{SEI}}\;$.

Depending on the mean molar volume of the formed SEI molecules, $V_{\text{SEI}}\;$, the SEI thickness grows in time by

(13) $$\frac{dL_{\text{SEI}}}{dt}=\frac{V_{\text{SEI}}}{F}j_{\text{SEI}}$$

The solutions of this equation for single‐transport processes are shown in S3, Supporting Information. Note that these solutions strongly differ in their SoC dependency.^[^ ^25^ ^]^

The proposed SEI growth mechanisms (Equation 7, 10, 11, 12) depend on many parameters. Often, two parameters appear as direct products a⋅b , making them indistinguishable for a fitting purpose. In these cases, we can choose one parameter to be fixed and optimize the other, granting a faster convergence. We showed in Section 4.1 that when optimizing both parameters simultaneously, they anticorrelate, as expected. Thus, our approach was perfectly valid. By fixing one parameter, each mechanism was characterized by a single parameter. As a consequence, the correlations between the parameters of the different mechanisms can also be interpreted as the relations between two different degradation mechanisms. The parameters of interest are $D_{e^{-}}\;$, κ , $D_{S}\;$, and $\kappa_{{\text{Li}}^{+}\text{,SEI}}\;$. To demonstrate that this does not limit the presented method, we analyzed a model with nongrouped parameters in S4, Supporting Information.

### 3.3 Experimental Data

As outlined in the previous section, one characteristic of the theoretical SEI degradation mechanisms is their dependence on the SoC. To investigate this dependence and possibly identify the actual SEI transport mechanisms, we performed an inverse analysis of the experimental data obtained by Keil et al.^[^ ^21^ ^]^ Here, a very brief outline of the experiment is given. In the experiment, they investigated the capacity loss of lithium‐ion batteries with a nickel–cobalt–aluminum oxide (NCA) cathode. They charged batteries to different initial SoCs and stored them for 9.5 months, performing check‐ups about every two months. As a result, they measured a clear trend of the capacity loss with time and SoC: the higher the initial SoC, the higher the capacity loss increasing in time. Notably, they detected a significant degradation (about 4 %) for 0 % initial SoC. By measuring the open‐circuit voltage *U* of the graphite anode, it is possible to perform inverse modeling of the measured capacity loss with the proposed degradation mechanisms.

### 3.4 Synthetic Data

To check whether EP‐BOLFI and BASQ work as intended, we produced synthetic data sets for which we know the occurring degradation mechanisms and their correct parameterization.

**Figure 2:**
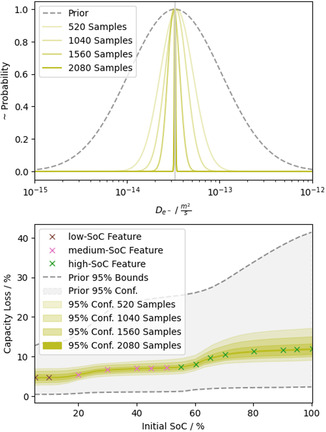
Exemplary evolution of one parameter's probability distribution (top panel) with simulated samples. The dashed line indicates the prior belief for this parameter. The colored lines represent the sequence of probability distributions after 520 simulated samples (transparent) up to 2080 samples (opaque). Note that the needed samples to get these results can be drastically reduced, as shown in Section 4.3. For better comparison, the probability distributions are normalized to one. The vertical gray line indicates the real parameter value for the synthetic data. In the lower panel, the corresponding 95% confidence areas of the parameterization are shown in data space. The crosses refer to the synthetic data points, and their coloring indicates their featurization into three segments: low SoC, medium SoC, and high SoC.

As a first synthetic dataset, we simulated SEI growth with the “Best Model” during the experimental storage protocol described in Section 3.3. Therefore, the synthetic storage data (see crosses in the lower panel of **Figure** **2**) is uniquely parameterized by the correct values for $D_{e^{-}}\;$ and $D_{S}\;$.

Additionally, we produced a second synthetic dataset to show the application of the methods on cycling data, which is a widespread experimental procedure. Further, we used this dataset to investigate EP‐BOLFI's performance under more difficult conditions and analyzed the impact of suitable feature choices on performance and obtained results. We generated the synthetic cycling data (see green line in Figure 7) by simulating SEI growth with the “Cycling Model” for 500 full cycles with 1C CC‐CV charge and 1C constant discharge. The corresponding voltage cut‐offs were 2.5 V and 4.2 V, with a cut‐off current of C/50. If noise does not exist, this data is uniquely parameterized with correct values for $D_{e^{-}}\;$, $D_{S}\;$, and $\kappa_{{\text{Li}}^{+}\text{,SEI}}\;$. We then applied different noise levels to this data for the analysis. To demonstrate that the presented method is not limited to synthetic data, we included an analysis of real cycling data^[^ ^49^ ^]^ in S5, Supporting Information.

## 4 Results & Discussion

In this section, we show the results of applying the presented Bayesian methods to perform inverse modeling of the degradation data with SEI growth. In the first subsection, we analyze storage data with EP‐BOLFI and BASQ to verify the methods and identify the dominant transport mechanism. In the second subsection, we show the results of using EP‐BOLFI to analyze cycling data, a ubiquitous experimental protocol. We investigate the impact of noise and the choice of features on the results. Finally, we compare our method to alternative approaches by solving the proposed inverse problems with available algorithms. Although the proposed problems are described by a simple parameter space, i.e., no local minima, we use EP‐BOLFI mainly with a very high dampening. The dampening slows the convergence and emulates realistic numbers of model evaluations needed for highly complex problems with multiple local minima. In the final comparison, we use EP‐BOLFI without dampening to show comparable numbers of model evaluations for simple problems. We discuss our findings along the way.

### 4.1 Storage Data

We first present the achieved results for synthetic storage data (see Section 3.4) to demonstrate the capability and credibility of the Bayesian methods. Afterwards, the results for real storage data (see Section 3.3) are shown and discussed.

For the inverse analysis of the storage data with EP‐BOLFI, features and a prior belief of the parameter range are needed. To enable correct identification of $D_{S}\;$ (see Equation 12), which is responsible for the low SoC contribution, and $D_{e^{-}}\;$ (see Equation 7) or κ (see Equation 10), which are responsible for the SoC‐dependent capacity loss, we choose the features to split the data into three SoC segments (see colored crosses in the lower panel). The 95% bounds of the prior belief for the parameters are chosen two orders of magnitudes around the correct/guessed value, such that the lower (higher) limit for each of the corresponding transport mechanisms would cause significantly less (more) degradation than observed in the data.

The results of the parameterization of the synthetic storage data with the “Best Model” are shown in Figure 2. The upper panel of Figure 2 shows the prior belief and sequential convergence of the probability distribution to the correct value with increasing knowledge (simulated samples), exemplary for $D_{e^{-}}\;$. In the lower panel of Figure 2, the simulations with the parameter values at the bounds of the sequential 95% areas for $D_{e^{-}}\;$ and $D_{S}\;$ are shown. Here, the colored areas refer to the 95% confidence areas of the joint probability distribution, again at different states of the algorithm. The more samples are analyzed, the narrower the confidence areas become around the synthetic data. Without considering noise, the data is uniquely parameterized by known values for $D_{e^{-}}\;$ and $D_{S}\;$. EP‐BOLFI finds the correct parameterization to describe the synthetic data perfectly. The uncertainty vanishes, and the solution converges within multiple thousand samples, even for a wide prior parameter space. Note that the needed samples can be drastically reduced by ideally adapting EP‐BOLFI's hyperparameters (e.g., weaker dampening).

**Figure 3:**
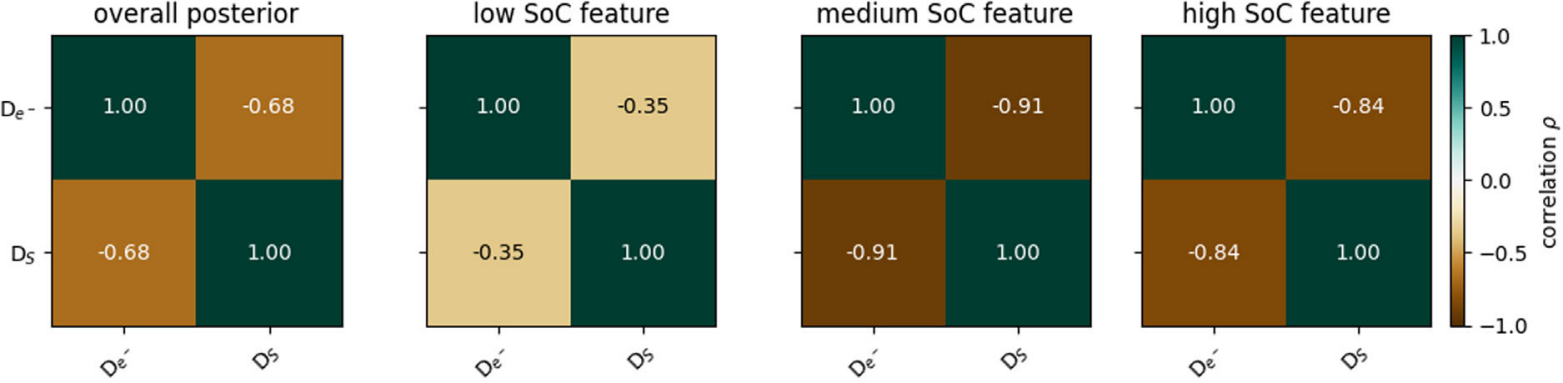
Correlation coefficients between the parameters $D_{e^{-}}\;\;$ and $D_{S}\;$ for the inverse modeling of the synthetic storage data with the “Best Model”. The leftmost panel shows the correlation in the overall posterior. The other panels refer to the feature‐specific correlations in the low, medium, and high SoC features. The color indicates the value of the correlation coefficients.

Besides the final parameterization, i.e., the means and variances of the final joint probability distribution, EP‐BOLFI also outputs the covariances. From these, the Pearson correlation coefficients ρ(X,Y) , with

(14) $$\rho (X,Y)=\frac{\text{cov}(X,Y)}{\sigma_{X}\sigma_{Y}}=\frac{\sum_{i=1}^{n}(X_{i}-\bar{X})(Y_{i}-\bar{Y})}{\sqrt{\sum_{i=1}^{n}{\left(X_{i}-\bar{X}\right)}^{2}}\sqrt{\sum_{i=1}^{n}{\left(Y_{i}-\bar{Y}\right)}^{2}}}$$

for the optimized parameters *X* and *Y* can be calculated. The correlation coefficient measures the linear connection between *X* and *Y*. For parameterization, the correlation between two parameters can be understood as to what degree parameter *X* has to be modified if *Y* changes to get a similar quality to fit the trend in the data. In reverse, the correlation coefficient contains the information on whether a specific parameter can be identified independently, i.e., has vanishing correlations to other parameters. As we describe each degradation mechanism with one parameter only, the correlation coefficient measures how identical or exchangeable these mechanisms are. **Figure** **3** shows the correlation coefficients between the parameters $D_{e^{-}}\;$ and $D_{S}\;$ for the overall posterior and the specific features obtained in the inverse modeling of the synthetic data. In the low SoC feature, the parameters show a low anti‐correlation, as solvent diffusion dominates the SEI growth. A strong anti‐correlation is achieved in the medium SoC feature, due to an almost equal contribution of electron diffusion and solvent diffusion. In the high SoC feature, the anti‐correlation is slightly reduced as electron diffusion dominates the SEI growth in this regime. The overall posterior yields an anti‐correlation of ρ=−0.68 , which is, in fact, a combination of the prior correlation and the correlations in the selected features. Therefore, the value lies between the others, as expected. For a more detailed analysis and discussion of feature‐specific correlations, see S6, Supporting Information.

**Figure 4:**
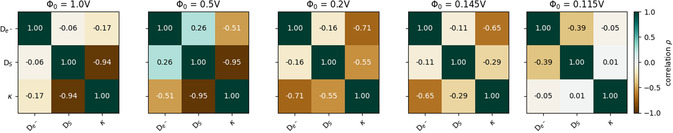
Correlation coefficients *ρ* between the parameters $D_{e^{-}}$, $D_{S}$, and κ  for the inverse modeling of the synthetic storage data with the “Overfitted Model”. The different panels correspond to five different values of the onset potential $\Phi_{0}\;$. From left to right, $\Phi_{0}\;$decreases, mimicking a transition of the electron conduction mechanism from SoC‐independent ($\Phi_{0}\in \{1.0V,0.5V\}\;$) to SoC‐dependent ($\Phi_{0}\in \{0.2V,0.145V\}\;$) to a vanishing contribution ($\Phi_{0}=0.115V\;$). The color indicates the value of the correlation coefficients.

To further investigate whether the obtained correlation values of the overall posterior reflect the physically expected interplay between the degradation mechanisms, we perform inverse modeling of the synthetic data with the “Overfitted Model”. By varying the value for $\Phi_{0}\;$, the electron conduction mechanism changes its SoC dependence. Note that experimental measures reveal that values for $\Phi_{0}\;$ lie between 0.8 V and 1.5 V.^[^ ^50^ ^]^ However, we also use lower values to mimic a similar SoC dependency as electron diffusion. **Figure** **4** shows the overall posterior correlations between $D_{e^{-}}\;$, $D_{S}\;$, and κ  for five different $\Phi_{0}\;$‐values, decreasing from left to right. $\rho (D_{S},\kappa )\;$ shows a direct anti‐correlation for high values of $\Phi_{0}\;$. It decreases for $\Phi_{0}<0.5\;$V and vanishes for $\Phi_{0}=0.115\;$V. Simultaneously, the anti‐correlation of $D_{e^{-}}\;$ and κ  increases to $\rho (D_{e^{-}},\kappa )=-0.71\;$ as the onset potential decreases to $\Phi_{0}=0.2\;$V. For lower values of $\Phi_{0}\;$, $\rho (D_{e^{-}},\kappa )\;$ decreases or vanishes. The obtained correlation values behave as expected. For $\Phi_{0}\in \{1.0V,0.5V\}\;$, electron conduction leads to almost constant capacity loss over the SoC range, similar to solvent diffusion. Therefore, a strong anti‐correlation is obtained for $\rho (D_{S},\kappa )\approx -1\;$, meaning these processes behave here very similarly, and only a weak anti‐correlation for $\rho (D_{e^{-}},\kappa )\;$. This changes for $\Phi_{0}\in \{0.2V,0.145V\}\;$ because then electron conduction contributes stronger and only for higher SoC‐values, becoming similar to electron diffusion. For $\Phi_{0}=0.115\;$V, electron conduction only contributes to the highest SoC points, resulting in vanishing overall correlation with the other mechanisms.

**Figure 5:**
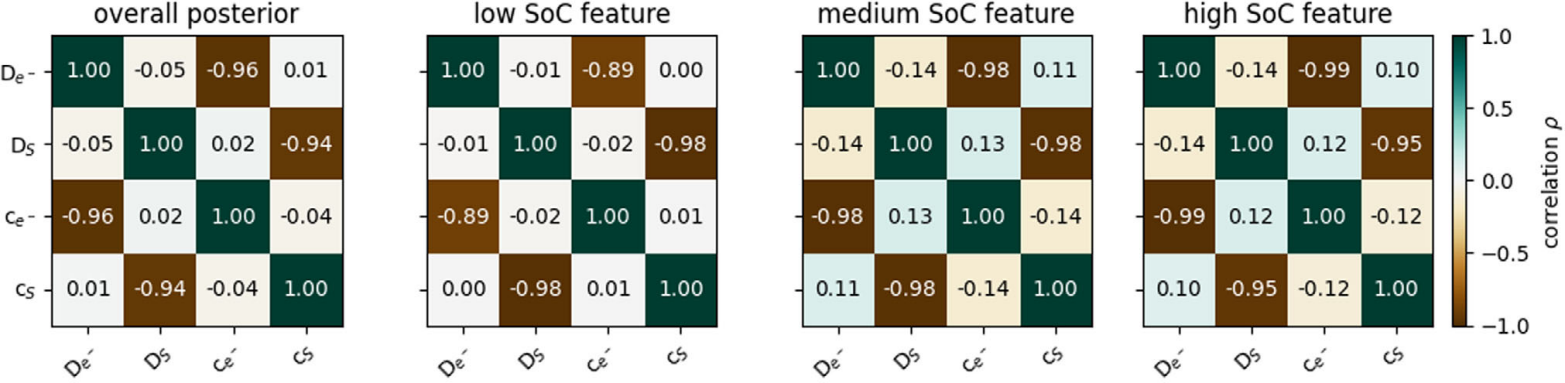
Correlation coefficients *ρ* between the parameters $D_{e^{-}}$, $D_{S}$, $c_{e^{-}}$, and $c_{S}\;$ for the inverse modeling of the synthetic storage data with the “Best Model”. The leftmost panel shows the correlations in the overall posterior. The other panels refer to the feature‐specific correlations in the low, medium, and high SoC features. The color indicates the value of the correlation coefficients.

Despite the interplay and exchangeability between the physical processes, the correlation values can also indicate an overparameterized model. To check if EP‐BOLFI can find lumped parameters, we perform inverse modeling of the synthetic data with the “Best Model.” Here, we optimize the parameters $D_{e^{-}}$, $D_{S}\;$, $c_{e^{-}}$, and $c_{S}\;$, where only the product of $D_{e^{-}}\;$ and $c_{e^{-}}\;$ (see Equation 7) and $D_{S}\;$ and $c_{S}\;$ (see Equation 12) are important. The resulting correlations are shown in **Figure** **5**. EP‐BOLFI finds a clear anti‐correlation between these parameter pairs ($\rho (c_{e^{-}},D_{e^{-}})\;$ and $\rho (c_{S},D_{S})\;$) in all features, as expected since the parameters appear as direct products (**Table** **1**).

**Table 1:** Mean model evidence calculated by BASQ for synthetic data and real storage data with and without penalty (due to a higher number of fit parameters). The number in bold indicate the model with the highest mean model evidence.

Models	Synt. data	Synt. data + penalty	Real data	Real data + penalty
“Best Model”	183.68	**178.67**	2.14	–**9.12**
“Wrong Model”	–130.19	–144.35	–282.19	–291.42
“Overfitted Model” ($\Phi_{0}=0.145\;$V)	**187.24**	168.34	**3.95**	–13.13

**Figure 6:**
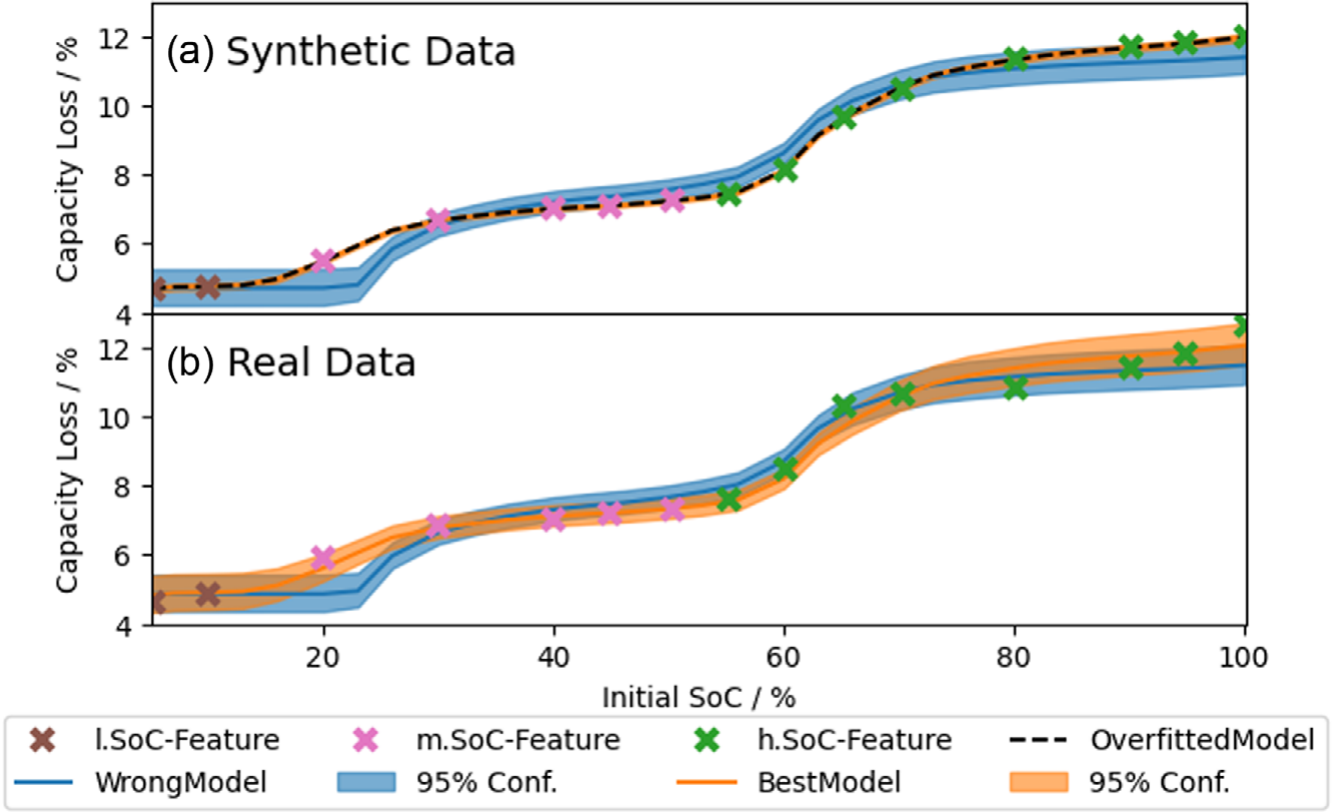
Results from performing inverse modeling of a) synthetic storage data and b) real storage data with the “Best Model” (orange) and “Wrong Model” (blue). The colored areas show the 95% confidence area of the parameterization. The colored crosses indicate the featurization of the data. The parameterization of the “Overfitted Model” with $\Phi_{0}=0.145\;$V (dashed black) is also shown for the synthetic storage data.

**Figure** **6** shows the results of the inverse modeling of the synthetic (upper panel) and real storage data (lower panel) with different models. The parameterized models “Best Model” (orange) and “Wrong Model” (blue) are shown with their corresponding 95% confidence intervals after equal sample numbers. In the upper panel, the final parameterization of the “Overfitted Model” (with $\Phi_{0}=0.145\;$V) is visualized by the dashed black line and coincides with the curve of the “Best Model”. Even though the correct model perfectly describes the synthetic data, the “Wrong Model” catches a similar trend in the SoC behavior. Without a mathematically reliable framework, it is difficult to evaluate which of the models fits the data the best. The parameterized models for the real storage data are shown in the lower panel. Again, both combinations follow the trend in the data reasonably well, and the corresponding 95% confidence areas include almost every data point. This demands a suitable model selection criterion that also considers the confidence areas.

Therefore, we use the BASQ algorithm to investigate the models, and its results are shown in Tab. I. The bold numbers indicate which model achieved the highest mean model evidence computed by BASQ in the different analyses. BASQ identifies the “Best Model” as better than the “Wrong Model” for the synthetic and real data. However, the “Overfitted Model” achieves slightly higher mean model evidence. As seen before, the best parameterization for the “Overfitted Model” and “Best Model” coincides visually (upper panel of Figure 6), so the higher evidence is assumed to be due to overfitting. Two options emerge to cope with the challenge of overparameterization: firstly, consider only reasonable parameter ranges. This would directly lead to the deselection of the models “Wrong Model” and “Overfitted Model” due to the nonphysical values of $\Phi_{0}\;$. The second option is introducing a penalty, which we pursue in this work (see S1, Supporting Information). This penalty lowers the likelihood of a certain parameter combination depending on the amount of parameters used in the model. Thus, a model with more optional parameters becomes less likely (i.e., achieves lower evidence) if it does not better fit the data. Then, the mean model evidence results, including a penalty, identify “Best Model” as the best model for synthetic data, as expected, and as the best model for the real storage data. Since solvent diffusion was used in this work to represent the SoC‐independent degradation and explain the capacity loss at low SoCs, BASQ identifies electron diffusion as the best transport mechanism to describe the SoC‐dependent characteristics in this storage data.

### 4.2 Cycling Data

In this subsection, the results for analyzing synthetic cycling data (see Section 3.4), as a prevalent experimental protocol, with EP‐BOLFI under more difficult conditions (e.g., significantly biased and uncertain prior belief, and noisy data) are shown. The obtained uncertainty, its dependence on noise ratio, and chosen featurization are investigated and discussed.

Again, EP‐BOLFI needs features and a prior belief for the parameters. For featurization, we have chosen a total of two different features. In the first feature, the data is fitted by a power law function ($y(t)\approx \alpha t^{\beta }\;$) to capture the correct contributions of the degradation mechanisms and thereby grant the proper trajectory of the capacity loss. Then, the first feature is $f_{1}={\left[\alpha ,\beta \right]}^{T}\;$. In the second feature, the capacity loss averaged over the last ten cycles is chosen $f_{2}=[\bar{\text{CL}}(t_{\text{av}})]\;$ to capture an absolute value of the capacity loss at the end of the cycling. The parameters’ prior distributions are biased by setting the means to values two to eight times larger than the correct values used for the synthetic data (see Table 2). The width of the prior distributions is chosen such that the 95% confidence bounds are one order of magnitude lower or higher, respectively, than the mean value.

**Table 2:** Comparison of parameter values for the inverse modeling of the synthetic cycling data. The true values correspond to the values used to produce the synthetic data. The mean prior values indicate the biased prior belief by placing the mean of the prior parameter distributions to bad "guessed" values. The final fit values are the means of the overall posterior distribution obtained after 4752 samples and coincide with the mean solution plotted in Figure 7. Note that the needed samples to get these results can be drastically reduced, as shown in Section 4.3.

Parameter	True value	Mean prior value	Final fit value
$D_{e^{-}}\;$	$3.3\cdot 10^{-14}\frac{m^{2}}{s}\;$	$7.0\cdot 10^{-14}\frac{m^{2}}{s}\;$	$3.36\cdot 10^{-14}\frac{m^{2}}{s}\;$
$D_{S}\;$	$2.5\cdot 10^{-21}\frac{m^{2}}{s}\;$	$8.0\cdot 10^{-21}\frac{m^{2}}{s}\;$	$2.53\cdot 10^{-21}\frac{m^{2}}{s}\;$
$\kappa_{{\text{Li}}^{+}\text{,SEI}}\;$	$1.0\cdot 10^{-6}\frac{S}{m}\;$	$8.0\cdot 10^{-6}\frac{S}{m}\;$	$0.99\cdot 10^{-6}\frac{S}{m}\;$

**Figure 7:**
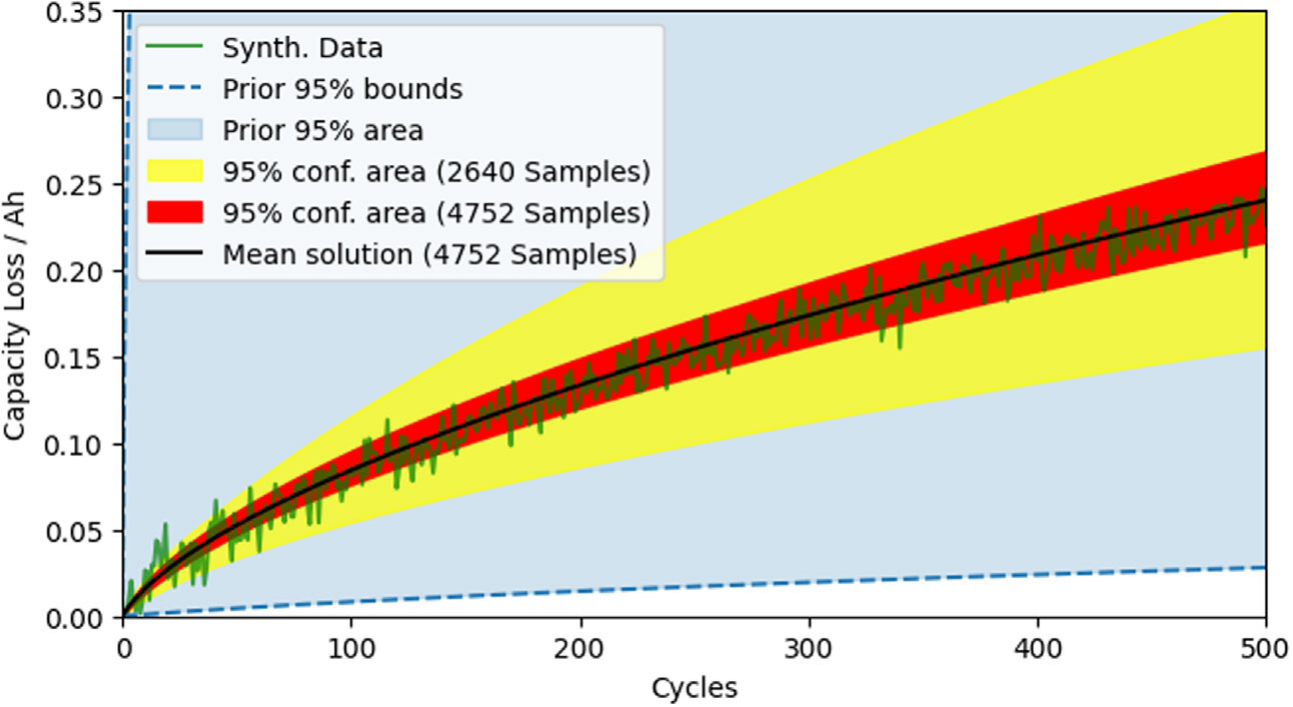
Results from performing inverse modeling of the synthetic data ($\sigma_{\text{noise}}^{2}=8\cdot 10^{-5}{\text{Ah}}^{2}\;$) with the correct “Cycling Model”. With an increasing number of drawn samples, the parameters’ joint probability distribution converges to the correct parameterization (black line) and a reasonable 95% confidence area (red) considering the noise level. The initial prior is wide (indicated by the blue 95% prior area) and significantly biased (nonsymmetric around the true values for the parameters).

**Figure** **7** shows the resulting sequential convergence in the parameterization of the synthetic cycling data (green line) with the correct “Cycling Model” for a relatively high noise level ($\sigma_{\text{noise}}^{2}=8\cdot 10^{-5}{\text{Ah}}^{2}\;$). The light blue area indicates the 95% confidence area of the initial prior belief. With increasing knowledge (simulated samples), the 95% confidence bounds of the joint probability hyper‐ellipsoid in data space (indicated by the colored areas) become symmetric around the data, and the uncertainty converges to a reasonable level. More simulated samples do not decrease the uncertainty significantly as it represents the noise in the data. E.g., the 95% confidence area after 6800 samples is very similar to the one after 4752 samples. **Table** **2** shows the parameter values for the simulated synthetic data, the initial prior means, and the final parameters. Note that these parameter values depend on other battery parameters and, therefore, should not be taken as absolute, i.e., a falsely assumed negative electrode surface area can change these parameters by orders of magnitude. The focus here is to show the correct identification of the “true” synthetic parameters. EP‐BOLFI detects a lower impact of solvent diffusion as initially guessed and, therefore, reduces $D_{S}\;$ to a value that shows an almost vanishing contribution of solvent diffusion. Equivalently, the value for $D_{e^{-}}\;$ is lowered to the correct value, which is a more difficult task since $D_{e^{-}}\;$ contributes to both $j_{\text{ED}}\;$ (Equation 7) and $j_{\text{EM}}\;$ (Equation 11). This means $D_{e^{-}}\;$ describes the correct contribution of a square root‐like capacity loss and influences additionally the linear capacity loss over time. A change in $D_{e^{-}}\;$ will affect both. However, $\kappa_{{\text{Li}}^{+}\text{,SEI}}\;$ affects the linear aspect only (see Equation 11). Identifying the right combination of $D_{e^{-}}\;$ and $\kappa_{{\text{Li}}^{+}\text{,SEI}}\;$ represents the correct interplay of a square root and linear SEI growth regime. The relative error of the parameters is below 2%, even for this noise level. One can see that even for a wrong initial guess, a very wide prior (blue area in Figure 7), and a significant noise level, a valid parameterization with reasonable uncertainty is found by EP‐BOLFI with a low number of samples.

**Table** **3** shows the posterior variances for the parameters of the “Cycling Model”, obtained for the inverse modeling of the synthetic cycling data with different applied noise levels. The lower the noise level, the lower the obtained parameter variances. The UQ of EP‐BOLFI indeed captures the noise and uncertainty in the data by the resulting confidence in the parameter space.

**Table 3:** Nondimensional posterior variances of the parameters for the “cycling model” after 6864 samples, obtained by performing inverse modeling of the synthetic cycling data with different applied noise levels. Note that the needed samples to get these results can be drastically reduced, as shown in Section 4.3. The variances are expressed in a logarithmic scale, i.e., the standard deviation measures the width of the distribution in e‐folds. This means the upper (lower) bounds of the *kσ*‐interval of parameter P are given by $P_{\pm }=P_{\text{mean}}\text{exp}(\pm k\sigma_{P})\;$. Therefore, comparing the variances shows the relative uncertainty in the specific parameter, which varies depending on the noise level. The prior belief of the parameters is equal for the different noise levels and symmetric around the true parameter values.

Variances	$\sigma_{\text{noise}}^{2}=8\cdot 10^{-6}{\text{Ah}}^{2}\;$	$\sigma_{\text{noise}}^{2}=8\cdot 10^{-5}{\text{Ah}}^{2}\;$
$\sigma_{D_{e^{-}}}^{2}\;$	0.01	0.08
$\sigma_{D_{S}}^{2}\;$	0.24	0.45
$\sigma_{\kappa_{{\text{Li}}^{+}\text{,SEI}}}^{2}\;$	0.02	0.13

**Figure 8:**
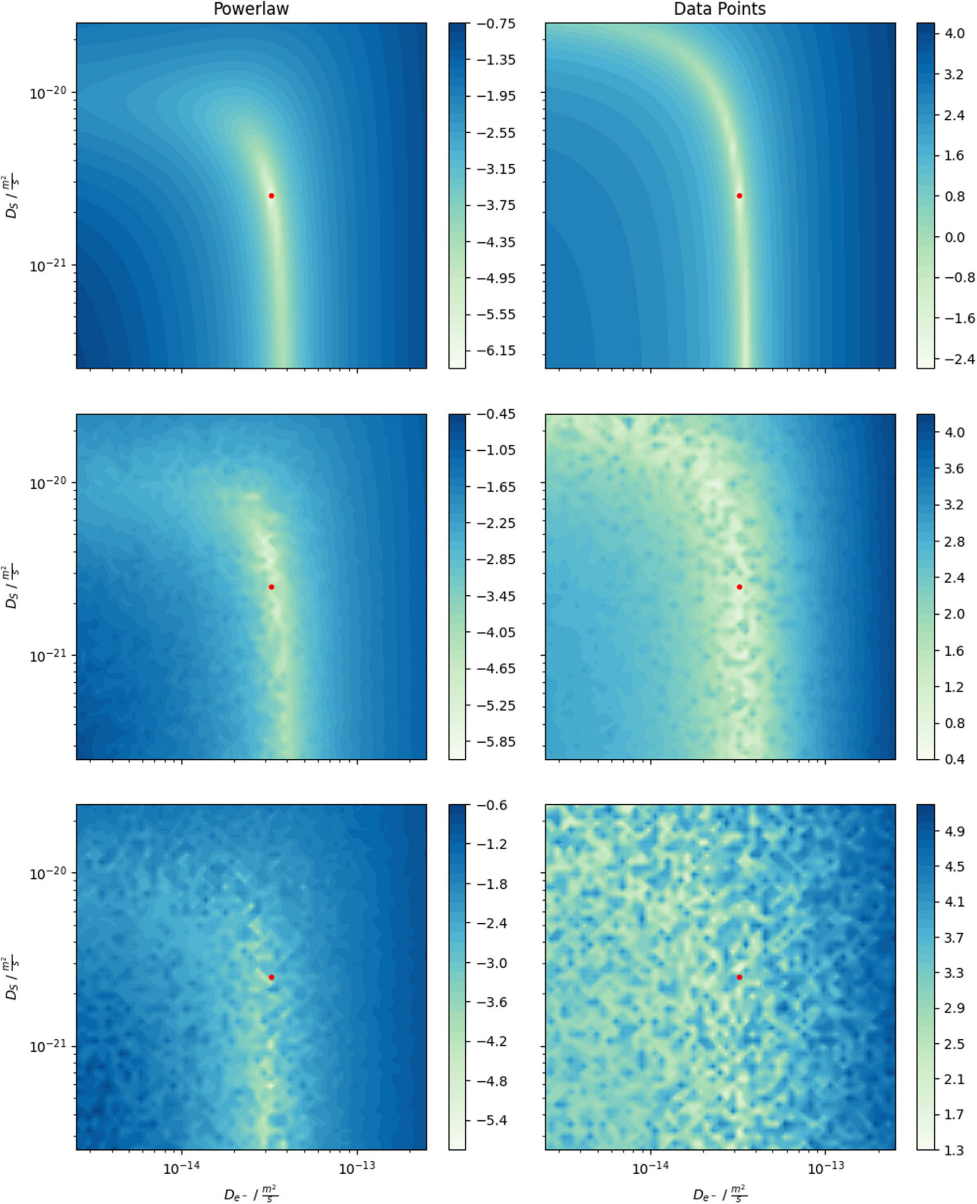
Landscape of a 2D parameter space ($D_{e^{-}}\;$–$D_{S}\;$) for different feature choices (columns) at various noise levels (top row: $\sigma_{\text{noise}}^{2}=0{\text{Ah}}^{2}\;$, middle row: $\sigma_{\text{noise}}^{2}=8\cdot 10^{-6}{\text{Ah}}^{2}\;$, bottom row: $\sigma_{\text{noise}}^{2}=8\cdot 10^{-5}{\text{Ah}}^{2}\;$). The red dots mark the true parameter configuration. In the left column, one power law through the capacity loss of 500 cycles is considered one feature ($f={\left[\alpha ,\beta \right]}^{T}\;$). In the right column, the data points of the 500 cycles without transformation are considered as one feature ($f={\left[\text{CL}(t_{\text{Cycle1}}),\ldots ,\text{CL}(t_{\text{Cycle500}})\right]}^{T}\;$). The color indicates the value of the loss function, which is given as the relative distance of the feature applied to the simulated and experimental data: $L=\text{log}\left(\left\|\frac{f_{i}\left(y_{\text{sim}}\left(\theta \right)\right)}{f_{i}\left(y_{\text{exp}}\right)}-1\right\|\right)\;$.

A central aspect of parameterization is how to measure the distance between data curves, e.g., experimental data and simulated data. The chosen transformations in the features act as such metrics because they assign each parameter combination a certain distance to the optimum (experimental data). Ideally, this metric can identify the correct parameter combination independently of the noise level. Therefore, we investigate a visualization of these metrics to show the impact of flexible feature choices due to EP. **Figure** **8** shows a two‐dimensional cross‐section of the landscape in parameter space for different features (columns) and noise levels (rows) at the correct value for $\kappa_{{\text{Li}}^{+}\text{,SEI}}\;$. The left column shows the results for a more suitable feature transformation (power law), whereas the right column shows the results for a basic feature (all data points) as a representative for classical approaches. The red dots indicate the true parameters. With vanishing noise, the landscape of the power law feature (upper left panel) shows a global minimum for the true parameters. Considering the data points (upper right panel) after each cycle as a feature (i.e., $f={\left[\text{CL}\left(t_{\text{Cycle1}}\right),\ldots ,\text{CL}\left(t_{\text{Cycle500}}\right)\right]}^{T}\;$), only a valley of minima can be identified rather than a global minimum. For higher noise levels, the data point feature shows a vast and smeared‐out valley of possible parameter combinations and is therefore considered a bad feature choice. In contrast, the power law feature maintains a narrow area around the correct parameters even for higher noise levels. Choosing certain features can be interpreted as inputting the visualized landscapes into the optimization algorithm. Therefore, a reasonable choice of features, their combinations, and noise resilience is crucial for correct parameterization, UQ, and correlations. Furthermore, they directly impact the convergence speed of the algorithm and the needed number of simulated samples. So, flexible adaptation of features through EP is a superior approach for parameterization purposes. In comparison, classical approaches, which use the data points as metric only, face a more challenging parameterization task. For a more detailed discussion of features, see S7, Supporting Information.

### 4.3 Comparison to Alternative Approaches

In this subsection, we compare the performance of different optimization algorithms to EP‐BOLFI on analyzing the proposed inverse problems.

We chose the least‐squares optimization algorithm within the SciPy library^[^ ^51^ ^]^ for a common standard approach. This method is similar to a gradient‐descent approach and is a local optimizer. We chose an MCMC algorithm implemented in the pymcmcstat package^[^ ^52^ ^]^ for a common Bayesian approach. The functionality of an MCMC algorithm is based on searching the parameter space with multiple Markov chains, whereby a singular chain can also be considered a local optimization. As a metric to assess the performance of an algorithm, we chose the number of model evaluations needed to get a reasonably good fit to the data. For the setup of the algorithms, the initial parameter guess needed for the least‐squares algorithm and the start of the MCMC chain align with the mean of the prior, which is used in EP‐BOLFI. So, the starting point of all algorithms is equivalent. As the presented optimization problems are simple, we use EP‐BOLFI without dampening in this section. Usually, the dampening prevents the algorithm from converging too quickly into local minima, maintaining its global optimization structure. This is turned off to obtain a better comparison to the other local optimizers.

**Table** **4** shows the results of analyzing the real storage data with the “Best Model” and the noisy synthetic cycling data with the “Cycling Model” with the different algorithms. In S8, Supporting Information, we include more details and a visualization of the comparison. In the case of the storage data, the least‐squares algorithm performs best and finds a reasonable fit in a few samples. Also, EP‐BOLFI, as a global optimizer, finds the correct parameter values within the first iteration of 20 samples. The uncertainty converged within the subsequent 20‐40 samples. The performance of the MCMC algorithm is more difficult to assess. The chain reached the correct area in parameter space at around 80 samples (see S8, Supporting Information). More samples in this area have to be simulated so that the chain gives the correct values for the means of the parameters, even after discarding the burn‐in samples of the chain. In addition to that, we tried different locations to start the chain. However, these chains did not find the correct parameter area under 100 samples. For a real MCMC analysis, multiple different chains should be set up. We noted that at least 80 samples are needed to find a reliable solution.

**Table 4:** Number of needed model evaluations to obtain a reasonable fit of the real storage data and noisy synthetic cycling data for different algorithms. In the case of MCMC simulations, it was not possible to give a fixed number that reliably indicates the needed model evaluations.

Data	Least‐squares	EP‐BOLFI	MCMC
Storage	10	20	>80
Cycling	38	32–96	>200

Regarding the cycling data, the least‐squares method finds the solution with about 38 samples. This optimization problem is slightly more difficult than the storage case. Depending on the initial guess of the parameters, we found that the least‐squares algorithm sometimes got stuck in local minima. Either the correct solution was found within a few samples, as the initial guess was close, or the optimization got stuck forever in the wrong area of parameter space. EP‐BOLFI found a good solution after the first iteration of 32 samples, which slightly improved to the correct solution after two subsequent iterations of 64 samples (see S8, Supporting Information). After one additional iteration (128 samples in total), the uncertainty converged and correct correlations emerged. The MCMC algorithm could not find the correct solution for the cycling data within 200 samples. The chain got stuck in a wrong parameter combination. Also, other starting points for the chains were not able to resolve the problem. A correct MCMC screening would require more chains covering the parameter space at an appropriate resolution.

## 5 Conclusion

In this work, we investigate physics‐based degradation models implemented in a full battery cell model with Bayesian ML algorithms. For ML algorithms to be applicable to complex physical models, the resulting parameterization and UQ must be correct and precise within the order of thousands of simulated samples. Moreover, correct ML‐based identification of the prevailing degradation model has to be achieved.

For synthetic storage data, the EP‐BOLFI algorithm can indeed find the correct parameterization and a reasonable corresponding uncertainty within a few samples. Further, valuable correlation values between the parameters are obtainable for the posterior distribution and self‐selected features. We show that these correlations correctly reflect the physical interplay between different processes and can show relevant processes in the chosen features.

By analyzing real storage data, we show that SEI growth by electron conduction and electron diffusion can describe a similar trend in capacity loss with increasing storage SoC. EP‐BOLFI can parameterize these models with reasonable uncertainty. To identify the best model for the experimental data, we investigate a Bayesian model selection criterion. This method consistently identifies the correct model and favors electron diffusion as the relevant SEI growth mechanism during battery storage for the investigated experimental data.

Further, we show that solving an inverse problem with noisy cycling data is possible for a significantly biased and uncertain prior belief. We identify the correct contribution and interplay of multiple SEI growth mechanisms. We visualize the importance of a suitable choice of features for convergence speed, obtained uncertainty, noise stability, and correct parameter identifiability. Further, we find that the resulting uncertainty correctly depends on the noise level in the data.

By comparing EP‐BOLFI to existing standard approaches, we show that it outperforms common MCMC algorithms and is about as fast as gradient‐descent methods. It provides further information (UQ and parameter correlations) with little additional effort. In addition, EP‐BOLFI is a global optimization algorithm built to tackle more complex problems as presented here.

To conclude this work, using sample‐efficient Bayesian algorithms enables the inverse modeling of real physics‐based models within acceptable computation time. The results contain correct parameterization with physically reliable UQ and summary statistics. We show that these methods identify electron diffusion responsible for the SoC‐dependent capacity loss during battery storage. However, for further confirmation, it is of major importance to analyze all‐encompassing experimental data measured at the most stable conditions and cell chemistries. Only then can the background of the SoC‐independent capacity loss, the influence of check‐ups, and the actual SEI growth mechanisms be identified.

To obtain the needed profound physical understanding of battery processes, we propose to perform inverse analysis of developed models with the presented methods to identify the correct physical process descriptions.

## Conflict of Interest

The authors declare no conflict of interest.

## Supporting information

Supplementary Material

## Data Availability

The data that support the findings of this study are available from the corresponding author upon reasonable request.
