# Supplementary material for: Physics‐Based Inverse Modeling of Battery Degradation with Bayesian Methods
Source: ChemSusChem. 2025 Jul 9;18(15):e202402336. doi: 10.1002/cssc.202402336 (PMC12302308; doi:10.1002/cssc.202402336)
Supplement: Supplementary file 1 — Supplementary Material [file CSSC-18-e202402336-s001.pdf]

Supporting Information to "Physics-based inverse modeling of battery degradation with Bayesian methods"

Micha C. J. Philipp, Yannick Kuhn, Arnulf Latz, and Birger Horstmann*
Institute for Engineering Thermodynamics, German Aerospace Center (DLR), Wilhelm-Runge-Straße 10, 89081 Ulm, Germany
Helmholtz Institute Ulm (HIU), Helmholtzstraße 11, 89081 Ulm, Germany and
Faculty of Natural Sciences, Ulm University, Albert-Einstein-Allee 11, 89081 Ulm, Germany

I. PENALTY IN BASQ

The likelihood LL used in this work originates from the implementation in BASQ [1]

$$LL = -\sum_{j=1}^{n} \log(2\pi R) - \frac{||y_{\text{exp}} - y_{\text{sim}}|| + \lambda \text{Tr}(\sum_{\text{Prior}}) \sum_{i=1} (\frac{\theta_i}{\theta_{i,0}})^2}{2R}, \tag{S1}$$

where n is the number of data points, R describes the noise in the data ($R = \exp(-\exp(\sigma^2_{\text{noise}})))$, λ is the parameter to adjust the weighting of the penalty, $\sum_{\text{Prior}}$ is the covariance matrix of the prior, θ_i is the value of parameter i and $\theta_{i,0}$ is the mean value of the prior of parameter i. For this work, we introduced the penalty term, which consists of the trace of the prior covariance matrix and parameter values themselves. Thereby, more available parameters are penalized by the trace of the covariance matrix, and the optimizing scheme is motivated to lower the parameter values as much as possible. In the used case, this is equivalent to Occam's razor since lowering the parameters as much as possible equals leaving out the corresponding effect. This penalty has to be calibrated by adjusting λ to not minimize the penalty alone but to find the optimal parameter configuration and still penalize more complex models.

II. SPME EQUATIONS AND CONDITIONS

In Table S1, the non-dimensional form of the governing equations of the SPMe model, as well as the equations for the terminal cell voltage, are listed. Note that the overbar indicates an electrode-averaged quantity. Boundary and initial conditions are needed to complete and solve the set of differential equations. These are shown in Tab. S2 and Tab. S3, respectively.

* birger.horstmann@dlr.de

III. TEMPORAL EVOLUTION OF THE SEI THICKNESS

By integrating $\frac{\mathrm{d}L_{\text{SEI}}}{\mathrm{d}t} = \frac{V_{\text{SEI}}}{F} j_{\text{SEI}}$ with the single current densities over time while keeping everything despite L_{SEI} constant, one can derive with the initial condition ($L_{\text{SEI}}(t = 0) = L_0$) a simplified evolution of the SEI thicknesses,

$$L_{\text{SEI,ED}} = \sqrt{2V_{\text{SEI}} c_{\text{e}^-} D_{\text{e}^-} \exp\left(-\tilde{\eta}_{\text{SEI}}\right) t + L_0^2}, \tag{S5a}$$

$$L_{\text{SEI,EC}} = \sqrt{2\frac{V_{\text{SEI}}}{F}\kappa(\Phi_0 - U(\text{SoC}))t + L_0^2}, \tag{S5b}$$

$$L_{\text{SEI,SD}} = \sqrt{2V_{\text{SEI}} c_{\text{S}} D_{\text{S}} t + L_0^2}, \tag{S5c}$$

$$L_{\text{SEI,EM}} = \frac{V_{\text{SEI}} c_{\text{e}^-} D_{\text{e}^-} F j_{\text{int}}}{2RT\kappa_{\text{Li}^+,\text{SEI}}} \exp\left(-\tilde{\eta}_{\text{SEI}}\right) t + L_0. \tag{S5d}$$

All storage mechanisms (eq. (S5a) - eq. (S5c)) can produce the same SEI growth behavior with the square root of time. From this perspective, the proposed storage mechanisms are not distinguishable. However, these mechanisms strongly differ in their dependency on the SoC. Where solvent diffusion is totally SoC-independent, electron conduction shows a slight SoC dependency, which can be varied by the chosen value for Φ_0. In contrast, electron diffusion predicts a strong exponential SoC-dependency through $\tilde{\eta}_{\text{SEI}}$.

IV. OPTIMIZING MULTIPLE NON-GROUPED PARAMETERS

As the proposed degradation mechanisms depend on many parameters that occur as products one is not able to distinguish these grouped parameters. Therefore, we kept one of these parameters fixed while optimizing the others. To show that the method is not limited to optimizing only one parameter per mechanism, we again performed the inverse modeling of the real storage data with the "Wrong Model". Here, we simultaneously optimize the parameters D_{S}, κ, and ϕ_0. Both, κ and ϕ_0, influence the EC transport mechanism, but are not directly grouped. In the main article we kept the latter fixed at $\Phi_0 = 0.145$V. Here we set the lower and upper 95% confidence bounds of the prior to 0.1V and 0.4V,

TABLE S1. Governing equations of SPMe battery model

particle radial diffusion	$\mathcal{C}_\mathrm{k}\partial_\mathrm{t} c^0_\mathrm{s,k} = -\frac{1}{r_\mathrm{k}^2}\partial_{\mathrm{r_k}}(r_\mathrm{k}^2\partial_{\mathrm{r_k}} c^0_\mathrm{s,k})$	(S2a)
electrolyte cation molar flux	$N^1_\mathrm{e,k} = -\epsilon^\mathrm{b}_\mathrm{k} D_\mathrm{e}(1)\partial_\mathrm{x} c^1_\mathrm{e,k} + \begin{cases} \frac{xt^+I}{\gamma_\mathrm{e}L_\mathrm{n}}, & \mathrm{k=n} \\ \frac{t^+I}{\gamma_\mathrm{e}}, & \mathrm{k=s} \\ \frac{(1-x)t^+I}{\gamma_\mathrm{e}L_\mathrm{p}}, & \mathrm{k=p} \end{cases}$	(S2b)
electrolyte cation diffusion	$\mathcal{C}_\mathrm{e}\epsilon_\mathrm{k}\gamma_\mathrm{e}\partial_\mathrm{t} c^1_\mathrm{e,k} = -\gamma_\mathrm{e}\partial_\mathrm{x} N^1_\mathrm{e,k} + \begin{cases} \frac{I}{L_\mathrm{n}}, & \mathrm{k=n} \\ 0, & \mathrm{k=s} \\ -\frac{I}{L_\mathrm{p}}, & \mathrm{k=p} \end{cases}$	(S2c)
terminal voltage	$V = \overline{U}_\mathrm{eq} + \overline{\eta}_\mathrm{r} + \overline{\eta}_\mathrm{c} + \overline{\Delta\Phi}_\mathrm{Elec} + \overline{\Delta\Phi}_\mathrm{Solid}$	(S2d)
equilibrium voltage	$\overline{U}_\mathrm{eq} = U_\mathrm{p}(c^0_\mathrm{s,p}\vert_{r_\mathrm{p}=1}) - U_\mathrm{n}(c^0_\mathrm{s,n}\vert_{r_\mathrm{n}=1})$	(S2e)
reaction overpotential	$\overline{\eta}_\mathrm{r} = -2\sinh^{-1}\left(\frac{I}{\overline{j}_\mathrm{0,p}L_\mathrm{p}}\right) - 2\sinh^{-1}\left(\frac{I}{\overline{j}_\mathrm{0,n}L_\mathrm{n}}\right)$	(S2f)
concentration overpotential	$\overline{\eta}_\mathrm{c} = 2\mathcal{C}_\mathrm{e}(1-t^+)(\overline{c}^1_\mathrm{e,p} - \overline{c}^1_\mathrm{e,n})$	(S2g)
negative electrode exchange current densities	$\overline{j}_\mathrm{0,n} = -\frac{1}{L_\mathrm{n}}\int_0^{L_\mathrm{n}} \frac{\gamma_\mathrm{n}}{\mathcal{C}_\mathrm{r,n}}(c^0_\mathrm{s,n})^{1/2}(1-c^0_\mathrm{s,n})^{1/2}(1+\mathcal{C}_\mathrm{e}c^1_\mathrm{s,n})^{1/2}\mathrm{d}x$	(S2h)
positive electrode exchange current densities	$\overline{j}_\mathrm{0,p} = -\frac{1}{L_\mathrm{p}}\int_{1-L_\mathrm{p}}^{L_\mathrm{p}} \frac{\gamma_\mathrm{p}}{\mathcal{C}_\mathrm{r,p}}(c^0_\mathrm{s,p})^{1/2}(1-c^0_\mathrm{s,p})^{1/2}(1+\mathcal{C}_\mathrm{e}c^1_\mathrm{s,p})^{1/2}\mathrm{d}x$	(S2i)
electrolyte ohmic losses	$\overline{\Delta\Phi}_\mathrm{Elec} = -\frac{I}{\hat{\kappa}_\mathrm{e}\kappa_\mathrm{e}(1)}\left(\frac{L_\mathrm{n}}{3\epsilon^\mathrm{b}_\mathrm{n}} + \frac{L_\mathrm{s}}{\epsilon^\mathrm{b}_\mathrm{s}} + \frac{L_\mathrm{p}}{3\epsilon^\mathrm{b}_\mathrm{p}}\right)$	(S2j)
solid phase ohmic losses	$\overline{\Delta\Phi}_\mathrm{Solid} = -\frac{I}{3}\left(\frac{L_\mathrm{p}}{\sigma_\mathrm{p}} - \frac{L_\mathrm{n}}{\sigma_\mathrm{n}}\right)$	(S2k)

TABLE S2. Boundary conditions

particle	$\partial_{\mathrm{r_k}} c^0_\mathrm{s,k}\vert_{r_k=0} = 0,\ -\frac{a_k\gamma_k}{\mathcal{C}_\mathrm{k}}\partial_{\mathrm{r_k}} = \begin{cases} \frac{I}{L_\mathrm{n}}, & \mathrm{k=n} \\ -\frac{I}{L_\mathrm{p}}, & \mathrm{k=p} \end{cases}$	(S3a)
molar flux	$N^1_\mathrm{e,n}\vert_{x=0} = 0,\ N^1_\mathrm{e,p}\vert_{x=1} = 0,\ N^1_\mathrm{e,n}\vert_{x=L_\mathrm{n}} = N^1_\mathrm{e,s}\vert_{x=L_\mathrm{n}},\ N^1_\mathrm{e,s}\vert_{x=1-L_\mathrm{p}} = N^1_\mathrm{e,p}\vert_{x=1-L_\mathrm{p}},$	(S3b)
concentration	$c^1_\mathrm{e,n}\vert_{x=L_\mathrm{n}} = c^1_\mathrm{e,s}\vert_{x=L_\mathrm{n}},\ c^1_\mathrm{e,s}\vert_{x=1-L_\mathrm{p}} = c^1_\mathrm{e,p}\vert_{x=1-L_\mathrm{p}}$	(S3c)

TABLE S3. Initial conditions

$c^0_\mathrm{s,k}(r_\mathrm{k}, 0) = c_\mathrm{k,0}$	(S4a)
$c^1_\mathrm{e,k}(x, 0) = 0$	(S4b)

respectively. The result after 2640 simulated samples is shown in Fig. S1. The mean value found is $\Phi_0 = 0.146$V which is similar to the fixed value. Note, that this value is not physically reliable. However, the overall result is also very similar to Fig. 6 in the main article. One can see that it is still possible to obtain very similar results within the same number of simulated samples. We conclude that the method is not limited to idealized parameterization tasks.

V. REAL EXPERIMENTAL CYCLING DATA

In the main article, we analyzed cycling data to investigate the capabilities of the Bayesian methods. We did this for synthetic data to check whether actual solutions were found. Here, we want to demonstrate that the method can be applied to real cycling data in the same way. As experimental data we use the degradation data from cycling a cell between SoC=0% and SoC=25% at $T = 40$C (see Fig. 9 from Zsoldos *et al.* [3]) Figure S2 shows the result of parameterizing the "Cycling Model" with EP-BOLFI for this cycling data after 1452 simulated samples. Again, the mean solution describes

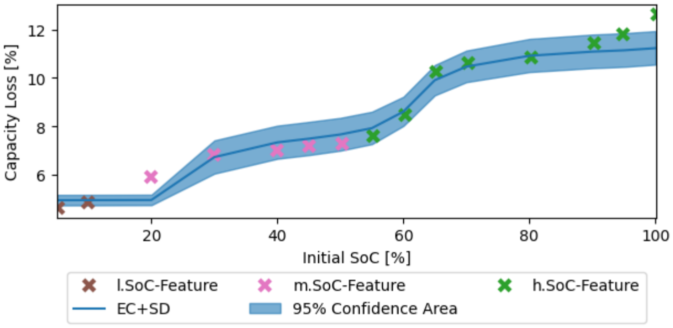

FIG. S1. Results from performing inverse modeling of real storage data [2] with the "Wrong Model" optimizing D_S, κ, and ϕ_0. The colored area shows the 95% confidence area of the parameterization. The colored crosses indicate the featurization of the data.

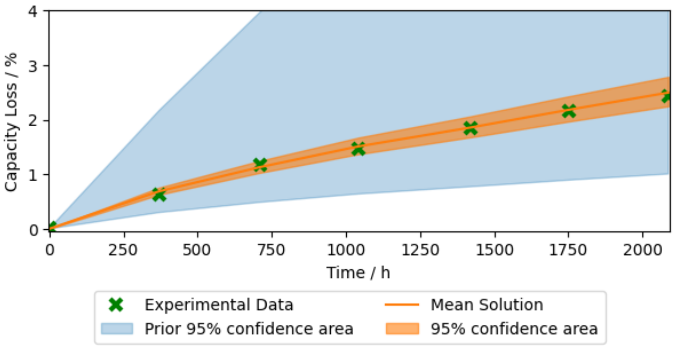

FIG. S2. Results from performing inverse modeling of real cycling data with the "Cycling Model" using the power law feature. The colored area shows the 95% confidence area of the parameterization. The colored crosses indicate the featurization of the data.

the data reasonably well. Note, that this does not allow direct physical conclusions, as many degradation effects may cause the observed capacity loss. The proposed "Cycling Model" is only one possibility that can describe the observations. For physical conclusions a thoroughly characterized battery cell is essential. However, this is beyond the scope of this work.

VI. FEATURE-SPECIFIC CORRELATIONS

In the EP-BOLFI's [4] structure, the feature-specific summary statistics become calculated backwards (by using $P(\theta \mid y) = \frac{P(y|\theta)P(\theta)}{P(y)}$) from the reduced posterior distribution, given the prior. For example, if a particular feature is analyzed, then the likelihood is estimated by a surrogate first. In the second step, the posterior is calculated by incorporating the prior belief. Once this posterior is reduced to a normal distribution, the likelihood can be calculated finally in the form of a normal distribution. The corresponding covariance matrix

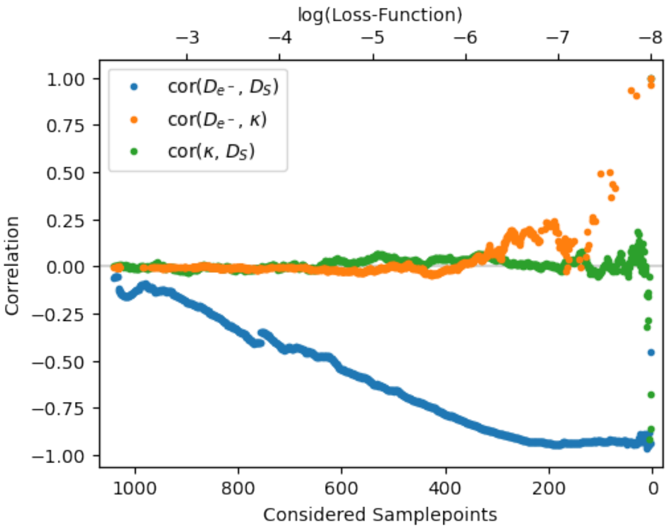

FIG. S3. Feature-specific correlations for the low-SoC-feature in the inverse modeling of the synthetic data with the "Overfitted Model" ($\Phi_0 = 0.145$V) and a total of 1040 samples. Each dot indicates the correlation value between two parameters when considering a certain number of samples. On the left, all 1040 drawn samples are considered. Moving to the right, the worst samples with the highest discrepancy to the synthetic data become left out. On the right, only the few best samples are considered.

for the simulated feature is then used to calculate the feature-specific correlations. However, in rare cases, this structure can cause meaningless values, as one can see by the following calculation. Assuming the prior and likelihood distribution to follow a normal distribution, i.e., $P(\theta) \sim \mathcal{N}(\mu, \sigma^2)$ and $P(y \mid \theta) \sim \mathcal{N}(\nu, \tau^2)$, one can show then that the posterior distribution also follows a normal distribution

$$\mathcal{N}(\rho^2(\frac{\mu}{\sigma^2} + \frac{\nu}{\tau^2}), \rho^2) \sim \mathcal{N}(\nu, \tau^2) \cdot \mathcal{N}(\mu, \sigma^2), \qquad \text{(S6)}$$

where $\rho^2 = \left(\frac{1}{\frac{1}{\sigma^2}+\frac{1}{\tau^2}}\right)$. If the posterior becomes more uncertain about a parameter distribution during the analysis of the feature likelihood, i.e., $\rho^2 > \sigma^2$, this can only be realized with $\tau^2 < 0$. Hence, no valuable summary statistics are available for this feature.

There are multiple ways still to get meaningful correlation values for a specific feature. One possibility is to perform the inverse modeling for the feature of interest only, using the already obtained parameterization from the entire run. Then, the posterior contains only the information from the specific feature and is always well-posed. It is important to note that the prior itself influences the result depending on the precision achieved in the feature. Therefore, enough samples with good precision have to be simulated to diminish the impact of the prior. Since EP-BOLFI uses the information carried by the best samples (see threshold ϵ

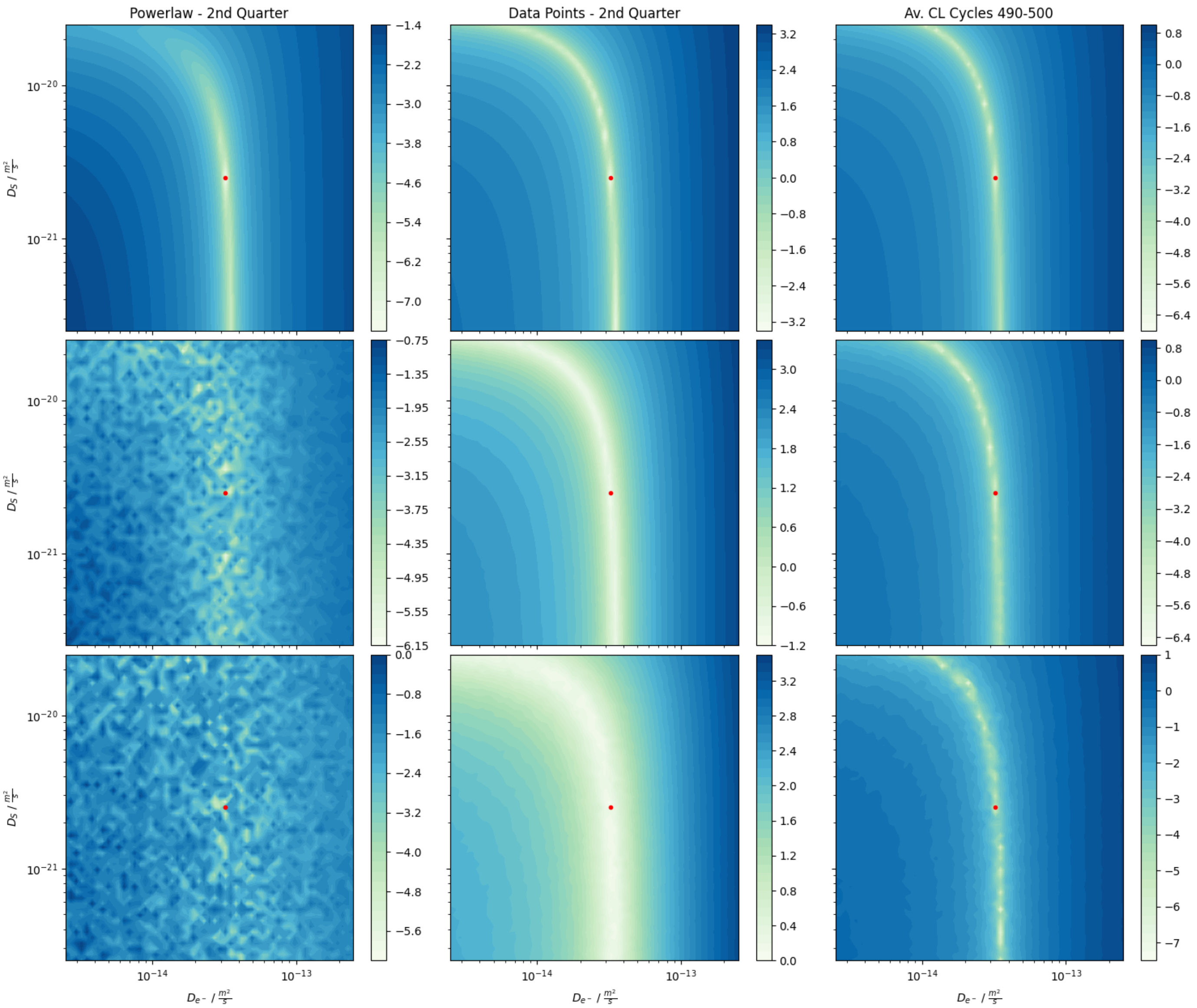

FIG. S4. Landscape of a two-dimensional parameter space for different feature choices (columns) at various noise levels (top row: $\sigma^2_{\text{noise}} = 0\text{Ah}^2$, middle row: $\sigma^2_{\text{noise}} = 8 \cdot 10^{-6}\text{Ah}^2$, bottom row: $\sigma^2_{\text{noise}} = 8 \cdot 10^{-5}\text{Ah}^2$). The red dots mark the true parameter configuration. In the left column, one power law through the capacity loss of a subset of cycles (cycles 126 to 250) is considered one feature ($f = [\alpha, \beta]^{\text{T}}$). This is in contrast to the left column of Fig. 8 in the main article, where the powerlaw is applied to all data points. In the middle column, the data points of cycles 126 to 250 without transformation are considered one feature ($f = [\text{CL}(t_{\text{Cycle 126}}), ..., \text{CL}(t_{\text{Cycle 250}})]^{\text{T}}$). In the right column, the average of the data points of the last ten cycles is considered as one feature ($f = [\overline{\text{CL}}(t_{\text{av}})]$). The color indicates the value of the loss function, which is given as the relative distance of the feature applied to the simulated and experimental data: $L = \log(||\frac{f_i(y_{\text{sim}}(\theta))}{f_i(y_{\text{exp}})} - 1||)$. This emphasizes that suitable feature-transformations and their noise resilience strongly depend on the underlying problem.

in $P(y \mid \theta) \sim P(\log(||f_i(y_{\text{sim}}(\theta)) - f_i(y_{\text{exp}})||) \leq \epsilon))$ to deduce the probability distribution, it is further crucial that enough samples achieve equal good precision as illustrated in Fig. S3. The data points were obtained by the inverse modeling of the synthetic storage data with the "Overfitted Model" (with 1040 samples) using only the low-SoC feature. The colored dots refer to the correlation values between the different model parameters in the low-SoC feature, depending on the number of considered samples. From left to right, the number of considered samples to calculate the correlation values decreases (lower x-axis), whereas the precision of the considered samples increases (upper x-axis). On the left side, all correlation values are close to zero, as all randomly drawn samples are considered. However, moving along the x-axis, only the samples that accurately de-

scribe the feature are considered, and real correlations emerge. Since electron conduction (with $\Phi_0 = 0.145$V) doesn't contribute to the low-SoC feature, the resulting correlation values with κ should be around zero, which is the case for most data points. This can change drastically if the number of considered samples is too low since the values for κ are drawn randomly and might show a positive or negative correlation by chance only (see orange and green dots for 0-200 sample points). Therefore, the second possibility to obtain reasonable correlations for a specific feature is to create this kind of correlation figures from the samples simulated in EP-BOLFI for the feature of interest.

VII. FEATURE-CHOICE

As the feature determines which metric is applied to measure the distance between the simulated and the experimental data, it directly influences the assigned likelihood for each drawn parameter configuration. The landscape of the loss function for the possible parameter combination visualizes this information. The existence of global minima refers to a unique optimal parameterization, whereas valleys indicate multiple optimal parameter combinations. The shape of such valleys contains information about the expected correlation values, which depend on the location in the parameter space. The width of the valleys represents the uncertainty in the parameterization. Different features capture different aspects of the data, e.g., power laws refer more to the actual shape of the curve in data space. In contrast, data points themselves, as a feature, try to minimize only the overall distance to the experimental data undisturbed by the actual shape. This can be seen in Fig. S4 as the feature for the averaged capacity loss of the last ten cycles shows a very sharp valley in the D_{e^-}-D_{S} plane. The main objective of this feature is to capture a single value of the CL at the end of the experiment. Therefore, it is not interested in the actual trajectory describing this capacity loss. Then, there are multiple solutions to tune electron diffusion, solvent diffusion, or electron migration to capture this point. However, not all possible solutions present the right trajectory, as the power law feature indicates only a subarea as optimal. The intersection of the minima of different features will yield the final parameterization. Suitable features vary enormously depending on the underlying optimization problem or differ even for one specific problem. For example, looking at power laws for small parts of noisy data only performs worse than considering the data points in the same parts (see lower panels of Fig. S4). In contrast, a power law through all data is less sensitive to noise than considering all data points (see Fig. 8 in the main article). Combining a smart choice of features, which captures different aspects of the data, enables a fast, correct and certain parameterization.

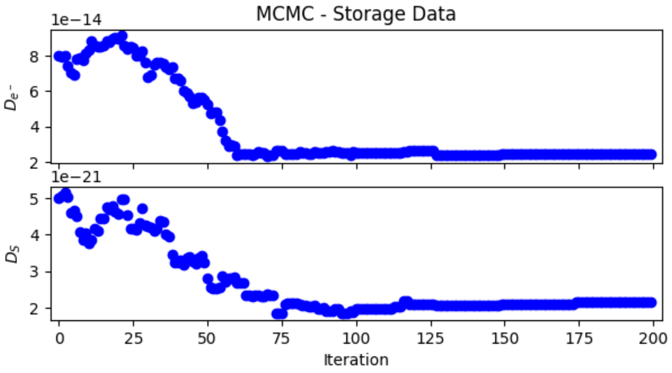

FIG. S5. Samples of the Markov chain obtained by analyzing the real storage data with an MCMC algorithm. The chain reaches the correct parameter area at about 80 samples. So that the mean of the chain is dominated by the proper area, the burn-in samples of the chain have to be removed, and/or more samples are needed.

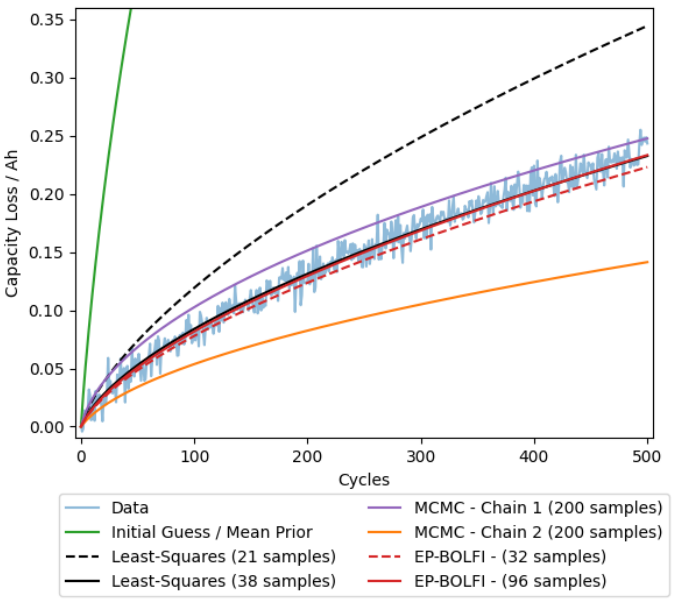

FIG. S6. Results of analyzing the noisy synthetic cycling data (bright blue line) with different algorithms. The green line indicates the initial guess.

VIII. COMPARISON TO ALTERNATIVE APPROACHES

To validate the potential of EP-BOLFI, we compared its performance to other known optimization algorithms by analyzing the real storage data and synthetic cycling data. The starting point is equivalent for all algorithms. Figure S5 shows the samples of the Markov chain obtained by analyzing the real storage data with an MCMC algorithm implemented in the pymcmcstat package [5]. One can see that the chain needs at least 50-80 samples to reach the correct parameter values, yielding a

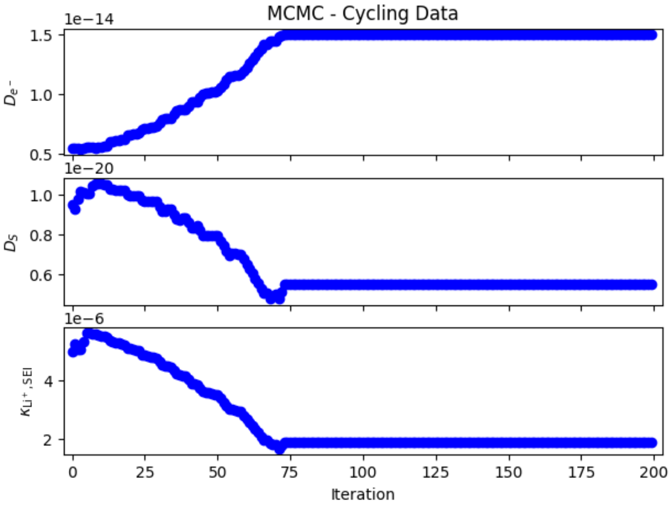

FIG. S7. Samples of the Markov chain obtained by analyzing the synthetic cycling data with an MCMC algorithm. The solution is shown as the orange line in Fig. S6.

wrong mean and variance of the chain up to this iteration. To get to the correct mean, one has to remove the first burn-in samples of the MCMC chain and consider more iterations in the correct parameter area. We conclude that at least 80 model evaluations are needed in this case. In addition, we tried to start the analysis with a different initial guess, with the result that the chains did not reach the correct parameter space within 100 samples. Figure S6 shows the results of analyzing the noisy synthetic cycling data with the different algorithms. The green line indicates the initial guess and is quite off. The least-squares algorithm within SciPy [6] behaves like a gradient-descent method approaching the correct solution starting from the initial guess. The solution after 21 evaluated samples (see dashed black line) is quite an improvement over the initial guess, and after 38 samples (see solid black line), the algorithm found a correct solution. In contrast, EP-BOLFI immediately gets a good fit after the first iteration of 32 samples (see dashed red line). With two more iterations (96 samples in total), the solution (see solid red line) coincides with the solution obtained by the least-squares algorithm. The solution of the MCMC algorithm after 200 samples with the equivalent initial guess is shown as the purple line and is still off the correct solution. The Markov chain got stuck on this solution after a few samples. Therefore, we started many chains at different initial guesses. The result was that either the initial guess was already reasonably fitting the data, or the chain got again stuck at a wrong position (see Fig. S7 and the orange line in Fig. S6 as the corresponding solution). One would need multiple chains at different locations to scan the parameter space for the optimal solution. We conclude that MCMC algorithms need more than 200 model evaluations.

[1] M. Adachi, Y. Kuhn, B. Horstmann, A. Latz, M. A. Osborne, and D. A. Howey, Bayesian model selection of lithium-ion battery models via bayesian quadrature, IFAC-PapersOnLine **56**, 10521 (2023).

[2] P. Keil, S. F. Schuster, J. Wilhelm, J. Travi, A. Hauser, R. C. Karl, and A. Jossen, Calendar aging of lithium-ion batteries, Journal of The Electrochemical Society **163**, A1872 (2016).

[3] E. S. Zsoldos, D. T. Thompson, W. Black, S. M. Azam, and J. R. Dahn, The operation window of lithium iron phosphate/graphite cells affects their lifetime, Journal of The Electrochemical Society **171**, 080527 (2024).

[4] Y. Kuhn, H. Wolf, A. Latz, and B. Horstmann, Bayesian parameterization of continuum battery models from featurized electrochemical measurements considering noise**, Batteries & Supercaps **6**, 10.1002/batt.202200374 (2023).

[5] P. Miles, pymcmcstat: A python package for bayesian inference using delayed rejection adaptive metropolis, Journal of Open Source Software **4**, 1417 (2019).

[6] P. Virtanen, R. Gommers, T. E. Oliphant, M. Haberland, T. Reddy, D. Cournapeau, E. Burovski, P. Peterson, W. Weckesser, J. Bright, S. J. van der Walt, M. Brett, J. Wilson, K. J. Millman, N. Mayorov, A. R. J. Nelson, E. Jones, R. Kern, E. Larson, C. J. Carey, İlhan Polat, Y. Feng, E. W. Moore, J. VanderPlas, D. Laxalde, J. Perktold, R. Cimrman, I. Henriksen, E. A. Quintero, C. R. Harris, A. M. Archibald, A. H. Ribeiro, F. Pedregosa, P. van Mulbregt, A. Vijaykumar, A. P. Bardelli, A. Rothberg, A. Hilboll, A. Kloeckner, A. Scopatz, A. Lee, A. Rokem, C. N. Woods, C. Fulton, C. Masson, C. Häggström, C. Fitzgerald, D. A. Nicholson, D. R. Hagen, D. V. Pasechnik, E. Olivetti, E. Martin, E. Wieser, F. Silva, F. Lenders, F. Wilhelm, G. Young, G. A. Price, G.-L. Ingold, G. E. Allen, G. R. Lee, H. Audren, I. Probst, J. P. Dietrich, J. Silterra, J. T. Webber, J. Slavič, J. Nothman, J. Buchner, J. Kulick, J. L. Schönberger, J. V. de Miranda Cardoso, J. Reimer, J. Harrington, J. L. C. Rodríguez, J. Nunez-Iglesias, J. Kuczynski, K. Tritz, M. Thoma, M. Newville, M. Kümmerer, M. Bolingbroke, M. Tartre, M. Pak, N. J. Smith, N. Nowaczyk, N. Shebanov, O. Pavlyk, P. A. Brodtkorb, P. Lee, R. T. McGibbon, R. Feldbauer, S. Lewis, S. Tygier, S. Sievert, S. Vigna, S. Peterson, S. More, T. Pudlik, T. Oshima, T. J. Pingel, T. P. Robitaille, T. Spura, T. R. Jones, T. Cera, T. Leslie, T. Zito, T. Krauss, U. Upadhyay, Y. O. Halchenko, and Y. Vázquez-Baeza, Scipy 1.0: fundamental algorithms for scientific computing in python, Nature Methods **17**, 261 (2020).
